# Supplementary material for: Health status and psychological outcomes after trauma: A prospective multicenter cohort study
Source: PLoS One. 2020 Apr 21;15(4):e0231649. doi: 10.1371/journal.pone.0231649 (PMC7173764; doi:10.1371/journal.pone.0231649)
Supplement: S1 Table — (DOCX) [file pone.0231649.s001.docx]

**S1 Table.** Injury group classification of the most common types of injury, based on the Abbreviated Injury Score [64].

| **Type of trauma** | **First three numbers of the AIS-code** | **Injury severity**  **(.1=minor, .6=maximal)** |
| --- | --- | --- |
| Pelvic injury | 856 | .2, .3, .4, .5 |
| Hip fracture | 853 | .3 |
| Tibia, complex foot or femur fracture | 854  857  858 | .2  .2  .2 |
| Shoulder and upper arm injury | 770  771  750  751 | .1, .2  .1, .2  .2  .2 |
| Radius, ulna or hand fracture | 752  753 | .1, .2, .3  .2 |
| Mild TBI* | 110  140  161 | .1, .2  .2  .1, .2 |
| Severe TBI** | 110  140  161 | .3  .3, .4, .5, .6  .3, .4, .5 |
| Facial fracture | 250  251 | .1, .2, .3  .1, .2, .3 |
| Thoracic injury | 441  419  442 | .1, .2, .3, .4, .5  .2, .3, .4, .5  .2, .3, .4, .5 |
| Rib fracture | 450 | .1, .2, .3, .4 |
| Mild abdominal injury | 516  510  521  530  540  541  542  543  544  545 | .1, .2  .1, .2  .2  .1  .1, .2  .2  .1, .2  .1, .2  .1, .2  .1, .2 |
| Severe abdominal injury | 516  510  520  520  521  540  541  542  543  544  545 | .3  .3  .3  .4, .5  .3, .4  .3, .4  .3, .4, .5  .3, .4, .5  .3, .4, .5  .3, .4, .5  .3, .4, .5 |
| Spinal cord injury | 640 | 3, .4, .5 |
| Stable vertebral fracture or disc injury | 650 | .2, .3 |

*Abbreviations: AIS, Abbreviated Injury Score; TBI, traumatic brain injury.*

** Concussion/ commotio cerebri, sequelae of intracranial injury. Sequelae of injury classifiable to S06.*

*** Traumatic cerebral oedema, focal brain injury, epidural haemorrhage, traumatic subdural heamorrhage, traumatic subarachnoid haemorrhage, intercranial injury unspecified, crushing injury of face, crushing injury of skull, crushing injury of other parts of head, crushing injury of head part unspecified, diffuse brain injury, intracranial injury with prolonged coma, other intracranial injuries traumatic haemorrhage (cerebellar/intracranial not specified).*
