# Supplementary material for: Health status and psychological outcomes after trauma: A prospective multicenter cohort study
Source: PLoS One. 2020 Apr 21;15(4):e0231649. doi: 10.1371/journal.pone.0231649 (PMC7173764; doi:10.1371/journal.pone.0231649)
Supplement: S2 Table — (DOCX) [file pone.0231649.s002.docx]

**S3 Table:** Missing sum scores of the original data and imputed data of the self-reported health status and psychological measures of the participants of the Brabant Injury Outcome Surveillance (n=4,883).

| **Questionnaire** | **1 week post-trauma**  **n (%)** | | **1 month post-trauma**  **n (%)** | | **3 months post-trauma**  **n (%)** | | **6 months post-trauma**  **n (%)** | | **12 months post-trauma**  **n (%)** | | **24 months post-trauma**  **n (%)** | |
| --- | --- | --- | --- | --- | --- | --- | --- | --- | --- | --- | --- | --- |
|  | *Original data* | *Imputed data* | *Original data* | *Imputed data* | *Original data* | *Imputed data* | *Original data* | *Imputed data* | *Original data* | *Imputed data* | *Original data* | *Imputed data* |
| **EQ-5D-3L*** | 3,192 (65.4%) | 3,107 (63.6%) | 2,085 (42.7%) | 1,908 (39.1%) | 1,909 (39.1%) | 1,671 (34.2%) | 1,594 (32.6%) | 1,293 (26.5%) | 1,807 (37.0%) | 1,468 (30.1%) | 2,173 (44.5%) | 2,141 (43.8%) |
| **HUI2**** | 3,282 (67.2%) | 3,107 (63.6%) | 2,276 (46.6%) | 1,912 (39.0%) | 2,427 (49.7%) | 2,148 (44.0%) | 2,686 (55.0%) | 2,441 (50.0%) | 2,721 (55.7%) | 2,492 (51.0%) | 2,832 (58.0%) | 2,746 (56.2%) |
| **HUI3**** | 3,289 (67.4%) | 3,107 (63.6%) | 2,253 (46.1%) | 1,912 (39.0%) | 2,417 (49.5%) | 2,148 (44.0%) | 2,678 (54.8%) | 2,441 (50.0%) | 2,709 (55.5%) | 2,492 (51.0%) | 2,824 (57.8%) | 2,746 (56.2%) |
| **HADSA***** | 2,727 (60.9%) | 2,704 (55.4%) | 1,913 (42.7%) | 1,836 (37.6%) | 2,125 (47.5%) | 2,054 (42.1%) | 2,358 (52.7%) | 2,285 (47.0%) | 2,375 (53.1%) | 2,314 (48.6%) | 2,824 (%) | 2,746 (56.2%) |
| **HADSD***** | 2,726 (60.9%) | 2,704 (55.4%) | 1,914 (42.8%) | 1,836 (37.6%) | 2,131 (47.6%) | 2,054 (42.1%) | 2,359 (52.7%) | 2,285 (47.0%) | 2,376 (53.1%) | 2,314 (48.6%) | 2,924 (59.9%) | 2,893 (59.2%) |
| **IES****** | 3,360 (68.8%) | 3,107 (63.6%) | 2,662 (54.5%) | 2,239 (45.9%) | 2,527 (51.8%) | 2,160 (44.2%) | 2,398 (49.1%) | 1,926 (39.4%) | 2,484 (50.9%) | 2,155 (44.1%) | 2,473 (50.7%) | 2,296 (47.0%) |

*Completed by the total study population. **Only administered in patients or proxy participants who completed the BIOS questionnaire. ***Only administered in patients who completed the BIOS questionnaire. ****Only administered in patients who completed the BIOS questionnaire or short questionnaire, except of patients aged ≥65 with a hip fracture who completed the short questionnaire.

*Abbreviations: EQ-5D-3L, EuroQol-5D-3L; HUI, Health Utilities Index; HADS, Hospital Anxiety and Depression Scale; IES, Impact of Event Scale.*
