# Supplementary material for: Health status and psychological outcomes after trauma: A prospective multicenter cohort study
Source: PLoS One. 2020 Apr 21;15(4):e0231649. doi: 10.1371/journal.pone.0231649 (PMC7173764; doi:10.1371/journal.pone.0231649)
Supplement: S1 File — (DOCX) [file pone.0231649.s003.docx]

**S2 File. Methods of imputed data of the Brabant Injury Outcome Surveillance**

Missing Abbreviated Injury Scale codes for the participants were manually checked in the electronic patient files, resulting in almost complete data for the ISS (0.9% missing). If at least half of the items of the Hospital Anxiety and Depression Scale (HADS) were completed, missing items were imputed using the individual subscale means according to the half-rule [65]. We assumed that missing values were missing at random (MAR) [66]. The imputation model included demographic and injury-related characteristics as well as summary scores of the questionnaires. Variables included in the imputation model were: name of the hospital in which the patient was admitted, age at the day of the trauma, gender, deceased during the study period, work prior to the trauma, patient aged ≥65 with a hip fracture, emotional well-being 1 day prior to the trauma (Health Utilities Index Mark 2 (HUI2) question 3 and Health Utilities Index Mark 3 (HUI3) question 6), the use of a walking aid 1 day prior to the trauma (HUI3 question 4), whether the questionnaires were completed by a proxy informant or not, cause of trauma, mode of transport to the hospital, hospital length of stay, Intensive Care Unit admission, comorbidities, Injury Severity Score, discharge destination, and the summary scores of the (pre-injury) EuroQol-5D-3L, (pre-injury) EuroQol Visual Analogue Scale, (pre-injury) Groningen Frailty Index, HUI2, HUI3, HADS subscale anxiety, HADS subscale depression, Impact of Event Scale, ICEpop CAPability measure for Older people and the Oxford Hip Score questionnaires collected at time points up until 1 year post-trauma. Imputed values for patients who did not participate at that specific follow-up questionnaire were back-transformed into missing values. Data of return to work was not imputed.
