# Supplementary material for: Health status and psychological outcomes after trauma: A prospective multicenter cohort study
Source: PLoS One. 2020 Apr 21;15(4):e0231649. doi: 10.1371/journal.pone.0231649 (PMC7173764; doi:10.1371/journal.pone.0231649)
Supplement: Attachment — Submitted filename: PONE-D-19-21191_R2_Editor comments.pdf [file pone.0231649.s006.pdf]

PLOS ONE

Health status and psychological outcomes after trauma; a prospective multicenter cohort study

--Manuscript Draft--

**Manuscript Number:**	PONE-D-19-21191R2
**Article Type:**	Research Article
**Full Title:**	Health status and psychological outcomes after trauma; a prospective multicenter cohort study
**Short Title:**	Health status and psychological outcomes after trauma
**Corresponding Author:**	Nena Kruithof<br>Elisabeth-TweeSteden Ziekenhuis<br>Tilburg, NETHERLANDS
**Keywords:**	injury; Trauma; hospitalization; longitudinal cohort study; health status; psychological outcome; Determinants; prognostic factors
**Abstract:**	Introduction: Survival after trauma has considerably improved. This warrants research on non-fatal outcome. We aimed to describe the recovery patterns of health status (HS) and psychological outcomes during 24 months of follow-up and to identify subgroups at risk of both short and long-term HS after trauma. Methods: Hospitalized patients with all types of injuries were included. Data were collected at 1 week 1, 3, 6, 12, and 24 months post-trauma. HS was assessed with the EuroQol-5D-3L (EQ-5D-3L) and the Health Utilities Index Mark 2 and 3 (HUI2/3). For the screening of symptoms of post-traumatic stress, anxiety and depression, the Impact of Event Scale (IES) and the Hospital Anxiety and Depression Scale (HADS) subscale anxiety (HADSA) and subscale depression (HADSD) were used. Recovery patterns of HS and psychological outcomes were examined with linear mixed model analyses. Results: A total of 4,883 patients participated (median age 68 (Interquartile range 53-80); 50% response rate). The mean (Standard Deviation (SD)) pre-injury EQ-5D-3L score was 0.85 (0.23). One week post-trauma, mean (SD) EQ-5D-3L, HUI2 and HUI3 scores were 0.49 (0.32), 0.61 (0.22) and 0.38 (0.31), respectively. These scores significantly improved to 0.77 (0.26), 0.77 (0.21) and 0.62 (0.35), respectively, at 24 months. Most recovery occurred up until 3 months. At long-term follow-up, patients of higher age, with comorbidities, longer hospital stay, lower extremity fracture and spine injury showed lower HS. The mean (SD) scores of the IES, HADSA and HADSD were respectively 14.80 (15.80), 4.92 (3.98) and 5.00 (4.28), respectively, at 1 week post-trauma and slightly improved over 24 months post-trauma to 10.35 (14.72), 4.31 (3.76) and 3.62 (3.87), respectively. Discussion: HS and psychological symptoms improved over time and most improvements occurred within 3 months post-trauma. The effect of severity and type of injury faded out over time. Patients frequently reported symptoms of post-traumatic stress.
**Order of Authors:**	Nena Kruithof
	Suzanne Polinder
	Leonie de Munter
	Cornelis van de Ree
	Koen Lansink
	Mariska de Jong
**Opposed Reviewers:**	Blerina Kellezi<br>Nottingham Trent University<br>Blerina.kellezi@ntu.ac.uk<br>Blerina recently published a comparable study.
	Helen Harcombe<br>Dunedin School of Medicine<br>helen.harcombe@otago.ac.nz<br>A few years ago, Helen published a comparable study.

Powered by Editorial Manager® and ProduXion Manager® from Aries Systems Corporation

Response to Reviewers:	Response to comments

Response to comments

Additional Editor Comments
-The additional explanation of SES needs to describe whether higher or lower scores indicate greater disadvantage to avoid potential mis-interpretation.
A: In the 'statistical analyses' paragraph, we described the following sentence: a lower status score indicates a lower SES whereas a higher status score indicates a higher SES. Furthermore, we described in the discussion and conclusion paragraphs that a lower status scores is an indicator of a lower SES-level (lines 425 and 473).

-The axes on Figure 5 are not clear. Which data do the titles on the left and right-hand x axes correspond to? Moreover, are these truly demonstrating "differences" or mean values? Ideally it is a good idea to depict variability (e.g., sd, se or 95%CI around the mean).
A: We changed Figure 3 as supposed by the editor. Furthermore, we made the description of the y-axis more clear.

-Supplementary Figure 5 does not appear to have been uploaded.
A: In this manuscript, there is no supplementary Figure 5. In the text, we did not refer to Supplementary Figure 5.

-The explanation of how to interpret a beta score is not sufficient. I suggest you follow the recommendation of Reviewer 2 in providing a clearer explanation of the beta value.
A: See the comment of reviewer 2: we have changed the sentence as supposed by the reviewer.

Comments reviewer 1
-There is still some language editing required which can be done at the proofing stage if the manuscript is accepted by the journal.
A: We did made several improvements in language editing throughout the manuscript.

-One sentence that is important to address in the abstract is: 'We aimed to describe the recovery patterns of health status (HS) and psychological outcomes during 24 months of follow-up and to identify subgroups at risk of both short and long-term HS after trauma'. The authors should make it clear that the focus is on identification of sub-groups at risk of poor health status.
A: In the abstract and manuscript, we now described: 'We aimed to 1) identify subgroups at risk of both short and long-term health status (HS) after trauma and 2) to describe the recovery patterns of HS and psychological outcomes during 24 months of follow-up.'

Comments reviewer 2
-The manuscript still requires significant editing for grammatical and spelling errors. For example, in line 71, no comma is required; line 106 should read 'compiles' and not 'complies'; line 140 should read 'comprise' and not 'comproise'. Errors are too extensive to list them all. Spelling/gramma correction software should help (not just the Word tool).
A: We have deleted the comma in line 71, we have changed the word 'complies' into 'compiles' and changed the word 'comproise' into 'comprise'. Furthermore, we made several improvements in language editing throughout the manuscript.

-Line 108. Please explain 'randomly controlled the data' in the data verification process more detail. All patient files or a sample? Did you check completeness, consistency, coherence and/or chronology?
A: We now described the following sentence: 'Quality of the data of the BTR and BIOS was checked on outliers and completeness by a trauma coordinator and researcher respectively. Furthermore, data from a sample of the trauma registry was checked manually by a trauma surgeon.'

-Line 240. Spell out FU. Again, using too many acronyms that are not widely recognized makes reading very arduous
A: We have spelled out the FU as 'follow-up' as suggested by the reviewer.

-Table 4 'Beta: measures how strong each predictor variable influences the dependent

	variable' could be replaced by 'mean increase in EQ-5D-3L score (improvement in quality of life) compared to the reference category.<br>A: We thank the reviewer for this suggestion. We changed the sentence as supposed by the reviewer.<br><br>Comments reviewer 3<br>-I am still a little confused by the status-scores.<br>A: In the 'statistical analyses' paragraph, we described the following sentence: a lower status score indicates a lower SES whereas a higher status score indicates a higher SES. Hopefully, this will lead to a better interpretation of the status score. Furthermore, we described in the discussion and conclusion paragraphs that a lower status scores is an indicator of a lower SES-level (lines 425 and 473). To interpret the results, we added the minimum and maximum scores.<br><br>-Some of the confidence intervals contains negative figures. What are the extremes of the scale? As this seems to be a Dutch scale, you could explain this better to international readers.<br>A: We added the minimum and maximum score of the EQ-5D-3L under Table 4.<br><br>-In the study design and participants section it is stated: "The Brabant Trauma Registry (BTR) complies pre-hospital and hospital data of all trauma patients admitted after presentation to the ED in the Noord-Brabant region." You probably intended to say compiles?<br>A: Indeed, we intented to say 'compile' instead of 'complies'. We changed the word 'complies' into 'compiles'.<br><br>Same paragraph: "Before the data of the BTR and data of the BIOS-study were merged, the researchers randomly controlled the data of the trauma registry." This is commendable, but what was "randomly"? 1 of 100, 1 of 1000, 20%? Please elaborate, otherwise this statement is not very descriptive.<br>A: We deleted the following text: 'Before the data of the BTR and data of the BIOS-study were merged, the researchers randomly controlled the data of the trauma registry'. We replaced this text with the following sentence: 'Quality of the data of the BTR and BIOS was checked on outliers and completeness by a trauma coordinator and researcher respectively. Furthermore, data from a sample of the trauma registry was checked manually by a trauma surgeon.'
**Additional Information:**	
**Question**	**Response**
**Financial Disclosure**<br><br>Enter a financial disclosure statement that describes the sources of funding for the work included in this submission. Review the submission guidelines for detailed requirements. View published research articles from *PLOS ONE* for specific examples.<br><br>This statement is required for submission and **will appear in the published article** if the submission is accepted. Please make sure it is accurate.	This work was supported by The Netherlands Organization for Health Research and Development (ZonMw) under grant number 80-84200-98-14225.<br><br>https://www.zonmw.nl/nl/<br><br>The funders had no role in study design, data collection and analysis, decision to publish, or preparation of the manuscript.

**Unfunded studies**<br>Enter: *The author(s) received no specific funding for this work.*<br><br>**Funded studies**<br>Enter a statement with the following details:<br>• Initials of the authors who received each award<br>• Grant numbers awarded to each author<br>• The full name of each funder<br>• URL of each funder website<br>• Did the sponsors or funders play any role in the study design, data collection and analysis, decision to publish, or preparation of the manuscript?<br>• **NO** - Include this sentence at the end of your statement: *The funders had no role in study design, data collection and analysis, decision to publish, or preparation of the manuscript.*<br>• **YES** - Specify the role(s) played.<br><br>* typeset	
**Competing Interests**<br><br>Use the instructions below to enter a competing interest statement for this submission. On behalf of all authors, disclose any competing interests that could be perceived to bias this work—acknowledging all financial support and any other relevant financial or non-financial competing interests.<br><br>This statement **will appear in the published article** if the submission is accepted. Please make sure it is accurate. View published research articles from *PLOS ONE* for specific examples.	The authors have declared that no competing interests exist.

**NO authors have competing interests**<br><br>Enter: *The authors have declared that no competing interests exist.*<br><br>**Authors with competing interests**<br><br>Enter competing interest details beginning with this statement:<br><br>*I have read the journal's policy and the authors of this manuscript have the following competing interests: [insert competing interests here]*<br><br>* typeset	
**Ethics Statement**<br><br>Enter an ethics statement for this submission. This statement is required if the study involved:<br><br>• Human participants<br>• Human specimens or tissue<br>• Vertebrate animals or cephalopods<br>• Vertebrate embryos or tissues<br>• Field research<br><br>Write "N/A" if the submission does not require an ethics statement.<br><br>General guidance is provided below. Consult the submission guidelines for detailed instructions. **Make sure that all information entered here is included in the Methods section of the manuscript.**	Ethics committee: Medical Ethics Committee Brabant (project number NL50258.028.14 and NW2016-09).<br><br>Prior to participation, participants signed an informed consent form.

Powered by Editorial Manager® and ProduXion Manager® from Aries Systems Corporation

**Format for specific study types**<br><br>**Human Subject Research (involving human participants and/or tissue)**<br>• Give the name of the institutional review board or ethics committee that approved the study<br>• Include the approval number and/or a statement indicating approval of this research<br>• Indicate the form of consent obtained (written/oral) or the reason that consent was not obtained (e.g. the data were analyzed anonymously)<br><br>**Animal Research (involving vertebrate animals, embryos or tissues)**<br>• Provide the name of the Institutional Animal Care and Use Committee (IACUC) or other relevant ethics board that reviewed the study protocol, and indicate whether they approved this research or granted a formal waiver of ethical approval<br>• Include an approval number if one was obtained<br>• If the study involved *non-human primates*, add *additional details* about animal welfare and steps taken to ameliorate suffering<br>• If anesthesia, euthanasia, or any kind of animal sacrifice is part of the study, include briefly which substances and/or methods were applied<br><br>**Field Research**<br><br>Include the following details if this study involves the collection of plant, animal, or other materials from a natural setting:<br>• Field permit number<br>• Name of the institution or relevant body that granted permission	
**Data Availability**<br><br>Authors are required to make all data underlying the findings described fully available, without restriction, and from the time of publication. PLOS allows rare exceptions to address legal and ethical concerns. See the PLOS Data Policy and FAQ for detailed information.	No - some restrictions will apply

A Data Availability Statement describing where the data can be found is required at submission. Your answers to this question constitute the Data Availability Statement and **will be published in the article**, if accepted.<br><br>**Important:** Stating 'data available on request from the author' is not sufficient. If your data are only available upon request, select 'No' for the first question and explain your exceptional situation in the text box.<br><br>Do the authors confirm that all data underlying the findings described in their manuscript are fully available without restriction?	
**Describe where the data may be found in full sentences. If you are copying our sample text, replace any instances of XXX with the appropriate details.**<br><br>• If the data are **held or will be held in a public repository**, include URLs, accession numbers or DOIs. If this information will only be available after acceptance, indicate this by ticking the box below. For example: *All XXX files are available from the XXX database (accession number(s) XXX, XXX.)*.<br>• If the data are all contained **within the manuscript and/or Supporting Information files**, enter the following: *All relevant data are within the manuscript and its Supporting Information files.*<br>• If neither of these applies but you are able to provide **details of access elsewhere**, with or without limitations, please do so. For example:<br><br>*Data cannot be shared publicly because of [XXX]. Data are available from the XXX Institutional Data Access / Ethics Committee (contact via XXX) for researchers who meet the criteria for access to confidential data.*<br><br>*The data underlying the results presented in the study are available from (include the name of the third party*	Data of the BIOS are unsuitable for public deposition due to the privacy of participant data. Data are anonymized, but due to relatively few severe cases, patients could be identified. Therefore, BIOS data are available for any interested researcher who meets the criteria for access to confidential data. The Brabant Trauma Registry (e-mail: secretariaat@nazb.nl) may be contacted to request data.

and contact information or URL).<br>• This text is appropriate if the data are owned by a third party and authors do not have permission to share the data.<br><br>* typeset	
Additional data availability information:	

N. Kruithof
Elisabeth-TweeSteden Hospital, Department Trauma TopCare
Hilvarenbeekseweg 60
5022 GC Tilburg, the Netherlands

20-03-2020

Dear Dr. M.J. Giummarra,

Please find enclosed the second revised version of our manuscript entitled ‘Health status and psychological outcomes after trauma: a prospective multicenter cohort study’ (PONE-D-19-21191). The text of the original manuscript has been modified entirely in line with the valuable comments and recommendations of you and the three reviewers. The changes will be addressed one by one in the addendum. We trust that the changes and improvements made this completely revised manuscript suitable for publication in PlosOne.

On behalf of all authors, I am looking forward to hearing from you.

Yours sincerely,

Nena Kruithof, PhD

Addendum: Response to comments

Manuscript

Click here to access/download;Manuscript;Manuscript_hersubmissie_V2_08

Health status and psychological outcomes after trauma; a prospective multicenter cohort study

Nena Kruithof[1*], Suzanne Polinder[2], Leonie de Munter[1], Cornelis L.P. van de Ree[1], Koen W.W. Lansink[1,3,4], Mariska A.C. de Jongh[1,4], BIOS-group

[1]ETZ Hospital (Elisabeth-TweeSteden Ziekenhuis), Department Trauma TopCare, Tilburg, the Netherlands

[2]Erasmus University Medical Centre, Department of Public Health, Rotterdam, the Netherlands

[3]ETZ Hospital (Elisabeth-TweeSteden Ziekenhuis), Department of Surgery, Tilburg, the Netherlands

[4]Brabant Trauma Registry, Network Emergency Care Brabant, Tilburg, the Netherlands

***Corresponding author:**

Email: nenakruithof@hotmail.com

Membership of the BIOS group is provided in the Acknowledgements

Abstract

Introduction: Survival after trauma has considerably improved. This warrants research on non-fatal outcome. We aimed to identify subgroups at risk of both short and long-term health status (HS) after trauma and to describe the recovery patterns of HS and psychological outcomes during 24 months of follow-up. **Methods:** Hospitalized patients with all types of injuries were included. Data were collected at 1 week 1, 3, 6, 12, and 24 months post-trauma. HS was assessed with the EuroQol-5D-3L (EQ-5D-3L) and the Health Utilities Index Mark 2 and 3 (HUI2/3). For the screening of symptoms of post-traumatic stress, anxiety and depression, the Impact of Event Scale (IES) and the Hospital Anxiety and Depression Scale (HADS) subscale anxiety (HADSA) and subscale depression (HADSD) were used. Recovery patterns of HS and psychological outcomes were examined with linear mixed model analyses. **Results:** A total of 4,883 patients participated (median age 68 (Interquartile range 53-80); 50% response rate). The mean (Standard Deviation (SD)) pre-injury EQ-5D-3L score was 0.85 (0.23). One week post-trauma, mean (SD) EQ-5D-3L, HUI2 and HUI3 scores were 0.49 (0.32), 0.61 (0.22) and 0.38 (0.31), respectively. These scores significantly improved to 0.77 (0.26), 0.77 (0.21) and 0.62 (0.35), respectively, at 24 months. Most recovery occurred up until 3 months. At long-term follow-up, patients of higher age, with comorbidities, longer hospital stay, lower extremity fracture and spine injury showed lower HS. The mean (SD) scores of the IES, HADSA and HADSD were respectively 14.80 (15.80), 4.92 (3.98) and 5.00 (4.28), respectively, at 1 week post-trauma and slightly improved over 24 months post-trauma to 10.35 (14.72), 4.31 (3.76) and 3.62 (3.87), respectively. **Discussion:** HS and psychological symptoms improved over time and most improvements occurred within 3 months post-trauma. The effects of severity and type of injury faded out over time. Patients frequently reported symptoms of post-traumatic stress.

Trail registration: ClinicalTrials.gov identifier: NCT02508675

Keywords: injury, trauma, hospitalization, longitudinal cohort study, health status, psychological outcome, determinants, prognostic factors

Introduction

Trauma poses a large burden on public health [1]. Reduction of trauma-related mortality in high-income countries [2] has resulted in increased numbers of trauma survivors with long-term injury impact [3], including reduced health status (HS) [4]. An improved understanding of the quality of survival of patients is critically important for improving health care quality and in evaluating trauma care. Furthermore, it is important to understand short and long-term recovery patterns of HS in terms of injured patient characteristics and to identify predictors of outcome of seriously injured patients [5, 6].

Establishing recovery patterns in the short and long-term requires longitudinal data [7]. Non-fatal outcomes after trauma can be assessed with an overall measure of HS. HS includes patients' physical functioning, state of mind and social activities [8]. In general, trauma has a large impact on HS [4, 9-12], but large variations between patients have been observed [11, 12].

In addition, several relevant non-fatal outcomes after trauma may be assessed more specifically. These include psychological outcomes, such as anxiety and depression. Psychological problems are often reported among trauma patients [13-18] and are associated with worse HS [9, 16].

The fact that trauma has an impact on diverse aspects of patient health, illustrates that a multidimensional approach is necessary for a comprehensive understanding of non-fatal outcomes after trauma. This also allows studying the mutual relations between non-fatal outcomes. Using a multidimensional approach to measure outcomes including HS and symptoms of depression, anxiety and post-traumatic stress will result in a comprehensive understanding of non-fatal outcomes after trauma. In addition, to assess prognostic factors for a poor outcome it is important to cover the entire spectrum of the trauma population without exclusion of particular patient groups (e.g. elderly). The number of longitudinal cohort studies that examine multiple non-fatal outcomes in a large sample with a broad inclusion of type and severity of injury is limited [12, 19-23]. Most studies start measuring outcomes at least 3 months after trauma, resulting in little knowledge about the real short-term consequences [11, 24-27].

The overall aim of the Brabant Injury Outcome Surveillance (BIOS), a population based longitudinal study, is to provide more insight into recovery patterns and determinants of non-fatal outcomes after trauma. The aims of this population-based study are 1. to identify prognostic factors for decreased short, mid and long-term HS and 2. to describe the 2 year recovery patterns of HS and

psychological outcome for different categories of trauma patients.. This information is important for understanding the short and long-term recovery patterns and for best informing provision of trauma care to injured patients with long-term disability.

Methods

Study design and participants

Data were obtained from the BIOS. The BIOS-study is a prospective observational cohort study in which HS and psychological outcomes are assessed in injured patients in the first 24 months after trauma. The methods of the BIOS have been described in detail in a published research protocol (doi: 10.1136/injuryprev-2016-042032) [28].

Recruitment occurred in all ten hospitals of the Noord-Brabant region (the Netherlands) from August 2015 until November 2016. Adults (≥18 years) who visited an emergency department ≤48 hours after trauma and who were admitted to the hospital, were invited to participate. All types of injuries were included regardless of the intent or severity of the injury. Patients who died between hospital discharge and the first week post-trauma, non-Dutch speaking patients, patients with no permanent address or patients with a pathological fracture were excluded. A proxy informant (caregiver or family member) was asked to complete the self-administered questionnaires if the patient was incapable of participating in the BIOS study him- or herself. Proxy informants were invited to enroll in the study 1 month post-trauma. Informal caregivers (e.g. family members) and paid caregivers (e.g. nurses) were allowed to function as proxy informants.

The Brabant Trauma Registry (BTR) compiles pre-hospital and hospital data of all trauma patients admitted after presentation to the ED in the Noord-Brabant region. Quality of the data of the BTR and BIOS was checked on outliers and completeness by a trauma coordinator and researcher respectively. Furthermore, data from a sample of the trauma registry was checked manually by a trauma surgeon.

The study was approved by the Medical Ethics Committee Brabant, the Netherlands (project numbers NL50258.028.14 and NW2016-09). Prior to participation, participants signed an informed consent form.

Data collection

Questionnaires were sent at 1 week and 1, 3, 6, 12 and 24 months after trauma. Based on the participants' preference, follow-up questionnaires were either completed by paper and pencil or digitally. The questionnaires collected data on general patient characteristics (date of birth, gender), self-reported comorbidities (by using a modified version of the Cumulative Illness Rating Scale [29]), self-reported HS (i.e. EuroQol-5D-3L (EQ-5D-3L) [30], the Health Utilities Index (HUI) Mark 2 and Mark 3 [31]) and self-reported psychological functioning (i.e., Hospital Anxiety and Depression Scale (HADS) [32] and the Impact of Event Scale (IES) [33]). Proxy informants did not complete questionnaires regarding psychological outcome.

To increase the response rate, patients who did not complete a questionnaire up until 3 or 6 months post-trauma were asked to complete a short version of the BIOS-questionnaire. Patients who completed the shortened questionnaire included those who could not be reached by phone and did not return a BIOS questionnaire. In this short questionnaire, educational level, comorbidities, the EQ-5D-3L and the IES were included.This short questionnaire did not include proxy assessments. In the shortened questionnaire, pre-injury HS was not collected.

If participants did not complete the questionnaire, they were not excluded from the study but they were still invited at the subsequent time points.

Outcome measures

The EQ-5D and HUI are used in various studies measuring HS after trauma [9, 11, 24, 26, 34-39]. The EQ-5D provides valid results for trauma patients when it is completed by a proxy informant [40]. Moreover, a combination of the EQ-5D and the HUI is recommended for use in trauma patients since the combination of these measures covers all relevant dimensions of health [37, 41].

The EQ-5D consists of the EQ-5D descriptive system and the EQ-visual analogue scale (EQ-VAS). The EQ-5D comprises? the following five dimensions: 'mobility', 'self-care', 'usual activities', 'pain/discomfort' and 'anxiety/depression'. Each dimension can be scored as 'no problems', 'moderate problems' or 'severe problems' [30]. A scoring algorithm is available by which each HS description can be expressed as a summary score. This summary score ranges from 0 for death and 1 for full health and can be interpreted as a judgment on the relative desirability of an HS compared with perfect health. A summary score of these five dimensions (EQ-5D utility) can be calculated by using the Dutch

tariffs [42]. The EQ-VAS is a vertical visual analogue scale with 0 indicating the worst imaginable health state and 100 indicating the best imaginable health state. The EQ-5D and EQ-VAS were also measured pre-injury, by asking participants 1 week or 1 month and proxy informants 1 month after the trauma for the patients' HS before sustaining the injury. The EQ-VAS was not included in the short questionnaire.

The HUI is a self-administered HS questionnaire that covers the main health domains that are affected by injury, with a particular focus on functional capacities. The HUI consists of 15 questions, classifying respondents into either the HUI2 or HUI3 health states [31]. Single-attribute and overall HS utility scores are calculated using the respective HUI2 and HUI3 utility functions. The results of the HUI questionnaires are converted by an algorithm into the levels of the complementary HUI2 and HUI3 classification system to form seven-element and eight-element health state vectors. From these vectors, single-attribute and overall health state utility scores are calculated [31].

For both the EQ-5D and the HUI, a scoring algorithm is used in which a score of 1 represents full health, 0 represents death and negative values indicate a HS of worse than death [30, 31].

The Hospital Anxiety and Depression Scale (HADS) was used to assess symptoms of anxiety and depression [32]. The HADS consists of 14 questions, 7 for symptoms of anxiety (HADSA) and 7 for depressive symptoms (HADSD). All questions have a 4-point response scale (0-3) and the scores for both subscales ranged from 0 to 21. A higher subscale score indicates greater severity of symptoms for anxiety and depression with a subscale value of ≥11 indicating a probable case (i.e., clinical symptoms) [32].

The IES was used to assess self-reported symptoms of post-traumatic stress [33]. The IES consists of 15 items of which the patient could use a 4-point scale (0=not at all, 1=rarely, 3=sometimes and 5=often) to determine whether the statement is present during the last seven days. The IES measures intrusive re-experience of the trauma and avoidance of trauma-related stimuli. A total sum score for the IES could be calculated ranging from 0 to 75. A score of ≥35 is considered as having symptoms of post-traumatic stress [33].

Prognostic factors

Hospital length of stay (LOS), admission to an Intensive Care Unit (ICU) and type and severity of injury were collected from the Brabant Trauma Registry and merged with the BIOS-data. The

Abbreviated Injury Scale (AIS) codes (AIS-90, update 2008) [43] were used to create 14 injury group classifications (e.g. hip fracture, severe abdominal injury) representing the most common types of injuries (see S1 Table). Patients who suffer multiple injuries could be classified into one or more injury group classifications.

Trauma severity was based on the Injury Severity Score (ISS). The ISS is based on the square of the highest Abbreviated Injury Scales (AIS) scores of the three most severely injured body regions with a range of 1 to 75. An ISS of ≥16 is considered severely injured [43].

To determine socio-economic status (SES), educational level was used. Educational level was categorized into three levels; low (primary education, preparatory secondary vocational education or without diploma), middle (university preparatory education, senior general secondary education or senior secondary vocational education and training), and high (academic degree or university of applied science).

Statistical analyses

Patients were included in the analyses if they completed a questionnaire for at least one of the predetermined time points. For the non-responders of the BIOS, we could not obtain educational level. Therefore, in the non-responders analysis, status scores from 2014 were used as a proxy to indicate SES. Status scores were based on the mean income, % of people with a low income, % of people with low educational level and % of unemployed people in the neighborhood. In 2014, the mean status score in the Netherlands was 0.28 [44]. A lower status score indicates a lower SES whereas a higher status score indicates a higher SES. Responders and non-responders were compared on age, gender, status score, ISS, type of trauma, LOS and admission to an ICU using Mann-Whitney U tests and Chi-square tests (χ^2).

Means and standard deviations (SDs) of the EQ-5D-3L, HUI2, HUI3, HADSD, HADSA and IES summary scores were calculated and reported for the total BIOS population and for the different subcategories.

Multiple imputation was conducted with the Multivariate Imputation by Chained Equations procedure [45] to handle missing baseline characteristics and sum scores of the questionnaires due to missing item scores (see S2). The dataset was imputed 15 times with 5 iterations. Sensitivity analysis

was performed in which only complete cases were included to compare results with the imputed datasets. S3 shows the differences between the original and imputed data.

Score options from each dimension of the EQ-5D were dichotomized into 0='no problems' and 1='moderate problems'/'severe problems'.

Four linear mixed models [46] with a random intercepts were performed to assess longitudinal association between prognostic factors and HS over the 24 months after trauma, which were divided into short-term (1 week and 1 month), mid-term (3 and 6 months) and long-term (12 and 24 months) associations. HS was measured with the EQ-5D-3L summary score.

The results were considered statistically significant at a level of $p<0.05$. All analyses were conducted in SPSS V.24 (Statistical Package for Social Sciences, Chicago, Illinois, USA), except of the multiple imputation which was performed in R version 3.4.0 (The R Project for Statistical Computing).

Results

BIOS cohort

During the inclusion period of the BIOS, a total of 10,227 patients were hospitalized because of trauma in one of the participating study centers. Patients were excluded if they did not speak the Dutch language (n=194), had no permanent address (n=32), died during their hospital stay within the first week after trauma (n=219) or had other reasons (n=8) (e.g., living abroad). Thus, 9,774 patients were eligible for participation in the BIOS, of whom 4,883 patients provided informed consent and were included (50% response rate). Of these 4,883 participants, 1,099 filled out the shortened questionnaires (see Fig 1).

At 1 week and 1, 3, 6, 12 and 24 months following the trauma, we collected data from of 1,776, 2,971, 3,109, 3,418, 3,105 and 2,734 participants (36.4%, 60.8%, 63.7%, 69.9%, 63.6% and 56.0%, respectively, of the study population) (see Fig 1). A total of 1,105 participants (22.6% of the study population) completed all BIOS questionnaires at each time point. In addition, data on pre-injury HS were obtained from 3,366 participants (69% of the study population). After the first week assessment, missing questionnaires were the result of non-response (i.e., patients who had provided no data at any of the previous time points) and loss to follow-up (i.e., patients who had provided data

for at least one of the previous time points). The main reason for participants to be lost to follow-up during the study period was that completing the questionnaires was too time consuming. Elderly, participants with low educational levels, longer hospital LOS, moderate injury (ISS 9-15), a hip fracture, severe traumatic brain injury (TBI) and those with severe abdominal trauma showed lower response rates to the 1 week questionnaire but provided data thereafter. In the BIOS, patients aged 18-24 and those who recovered completely were most likely to be lost to follow-up.

-insert Fig1 here-

Study population

The median age of the study population was 68 years (IQR 53-80) (Table 1). Responders had a median ISS of 5 (IQR [4-9]) and a large part of the population reported comorbidities. Mild TBI (27.1%) and hip fracture (25.9%) were the most common types of trauma among the participants included in the BIOS. The majority of the participants (n=2,562, 52.5%) had a low educational level. A total of 407 participants (8% of the study population) were represented by a proxy informant.

Compared to the non-responders, participants were more severely injured and had a higher probability of being admitted to the ICU. In addition, responders had a higher median status score (based on the mean income, % of people with a low income, % of people with low educational level and % of unemployed people in the neighborhood) compared to the general Dutch population (mean 0.28) and compared to the median status score of the non-responders (median 0.33, min. score -3.03, max. score 2,58). Patients aged 18-44 and ≥85 years showed relatively low response rates (35%-40% and 39%, respectively). Patients with minor injuries (ISS 1-3) revealed a low response rate (46%), as well as patients with a hospital LOS of ≤2 or ≥15 days (46% and 45%, respectively).

Table 1. Characteristics of responders and non-responders of the Brabant Injury Outcome Surveillance.

Characteristics	Responders (n=4,883) %	Non-responders (n=4,891) %
**Gender (male)**	2,329 (47.7%)	2,407 (49.0%)
**Median age (yrs)**	68 (IQR 53-80)	70 (IQR 46-84)
18-24	217 (4.4%)	400 (8.2%)
25-44	516 (10.6%)	767 (15.7%)
45-64	1,364 (27.9%)	1,006 (20.6%)
65-74	963 (19.7%)	563 (11.5%)
75-84	1,102 (22.6%)	1,030 (21.1%)
≥85	721 (14.8%)	1,125 (23.0%)
Missing	0 (0.0%)	0 (0.0%)
**Median SES status score**	0.33 (IQR -0.24-0.84)	0.13 (IQR -0.36-0.73)
Missing	60 (1.2%)	68 (1.4%)
**Median days hospital LOS**	4 (IQR 2-8)	4 (IQR 2-8)

≤2	1,325 (27.1%)	1,528 (31.2%)
3-7	1,944 (39.8%)	1,642 (33.6%)
8-14	937 (19.2%)	911 (18.6%)
≥15	346 (7.1%)	421 (8.6%)
Missing	331 (6.8%)	389 (8.0%)
**Type of injury**		
Pelvic injury	293 (6.0%)	151 (3.1%)
Hip fracture	1,266 (25.9%)	1,099 (22.5%)
Tibia, complex foot or femur fracture	569 (11.7%)	505 (10.3%)
Shoulder and upper arm injury	473 (9.7%)	417 (8.5%)
Radius, ulna or hand fracture	308 (6.3%)	283 (5.8%)
Mild TBI	1,324 (27.1%)	1,443 (29.5%)
Serious TBI	126 (2.6%)	130 (2.7)
Severe TBI	77 (1.6%)	77 (1.6)
Facial fracture	249 (5.1%)	303 (6.2%)
Thoracic injury	198 (4.1%)	162 (3.3%)
Rib fracture	541 (11.1%)	398 (8.1%)
Mild abdominal injury	87 (1.8%)	89 (1.8%)
Severe abdominal injury	36 (0.7%)	30 (0.6%)
Spinal cord injury	27 (0.6%)	10 (0.2%)
Stable vertebral fracture or disc injury	301 (6.2%)	249 (5.1%)
**Injury severity**	5 (IQR 4-9)	5 (IQR 2-9)
ISS 1-3	1,145 (23.4%)	1,360 (27.8%)
ISS 4-8	1,597 (32.7%)	1,320 (27.0%)
ISS 9-15	1,857 (38.0%)	1,627 (33.3%)
ISS ≥16	239 (4.9%)	194 (4.0%)
Missing	45 (0.9%)	390 (8.0%)
**ICU-admission (yes)**	358 (7.3%)	292 (6.0%)
Missing	0 (0.0%)	0 (0.0%)

Abbreviations:SES, social-economic status; ICU, intensive care unit; ISS, Injury Severity Score; IQR, Interquartile range; LOS, length of stay; TBI, traumatic brain injury; yrs, years.

Health status

The mean EQ-5D-3L summary score increased from 0.49 (SD 0.32) at 1 week post-trauma to 0.77 (SD 0.26) at 24 months post-trauma. The mean pre-injury EQ-5D score was 0.85 (SD 0.23). In addition, the mean (SD) HUI2 and HUI3 scores increased from 0.61 (0.22) and 0.38 (0.31) at 1 week post-trauma to 0.77 (0.21) and 0.62 (0.35) at 24 months post-trauma, respectively (see Table 2). With regard to the individual domains of the EQ-5D, trauma patients reported various problems on the 'mobility, 'usual activities' and 'pain/discomfort' dimensions during the 24 months of follow-up (see Fig 2). In addition, during the 24 months, the prevalence of problems on all dimensions of the EQ-5D decreased, but remained higher at 24 months compared to pre-injury (46% and 32%, respectively for mobility, 23% and 16%, respectively for self-care, 44% and 26%, respectively for usual activities, 52% and 32%, respectively for pain/discomfort and 22% and 16%, respectively for anxiety/depression).

Table 2. Mean (SD) summary scores of self-reported health status and psychological outcomes up to 2 years post-trauma.

Time post-trauma	EQ-5D-3L*		HUI2**		HUI3**		HADSA***		HADSD***		IES****	
	N	Mean (SD)	N	Mean (SD)	N	Mean (SD)	N	Mean (SD)	N	Mean (SD)	N	Mean (SD)
1 week	1,776	0.49 (0.32)	1,776	0.61 (0.22)	1,776	0.38 (0.31)	1,776	4.92 (3.98)	1,776	5.00 (4.28)	1,776	14.80 (15.80)
1 month	2,97	0.56	2,97	0.67	2,97	0.45	2,64	4.81	2,64	4.77	2,64	14.44

	1	(0.30)	1	(0.22)	1	(0.35)	4	(3.95)	4	(4.17)	4	(15.73)
3 months	3,131	0.69 (0.27)	2,735	0.72 (0.22)	2,735	0.53 (0.35)	2,423	4.57 (3.80)	2,423	4.24 (4.02)	2,819	12.75 (15.47)
6 months	3,433	0.74 (0.25)	2,442	0.75 (0.22)	2,442	0.58 (0.35)	2,191	4.21 (3.79)	2,191	3.91 (4.01)	3,182	11.42 (15.28)
12 months	3,151	0.76 (0.25)	2,391	0.76 (0.22)	2,391	0.60 (0.36)	2,165	4.32 (3.78)	2,165	3.74 (3.97)	2,925	10.98 (14.98)
24 months	2,734	0.77 (0.26)	2,137	0.77 (0.21)	2,137	0.62 (0.35)	1,990	4.31 (3.76)	1,990	3.62 (3.87)	2,587	10.35 (14.72)

*Completed by total study population. **Not administered to patients who completed only the short questionnaire. ***Not administered to proxy participants or to patients who completed only the short questionnaire. ****Not administered to patients aged ≥65 with a hip fracture who completed only the short questionnaire.
Range of EQ-5D-3L: 0-1. Mean EQ-5D-3L of the general Dutch population: 0.87 [47].
Range of HUI 2/3: 0-1
Range of HADSA and HADSD: 0-21
Range of IES: 0-75
Abbreviations: SD, standard deviation; EQ-5D, EuroQol-5D-3L; HUI2/3, Health Utilities Index Mark 2/3; HADS, Hospital Anxiety and Depression Scale; HADSA, Hospital Anxiety and Depression scale, subscale anxiety; HADSD, Hospital Anxiety and Depression scale, subscale depression; IES, Impact of Event Scale.

-insert Fig2 here-

Psychological outcomes

The mean summary scores (SD) of the HADSA (clinical symptoms of anxiety), HADSD (clinical symptoms of depression) and IES (symptoms of post-traumatic stress) slightly decreased from 4.92 (3.98), 5.00 (4.28) and 14.80 (15.80), respectively, at 1 week post-trauma to 4.31 (3.76), 3.62 (3.87) and 10.35 (14.72), respectively, at 24 months post-trauma (see Table 2).

The prevalence rates of clinical symptoms of anxiety (HADSA≥11), depression (HADSD≥11) and post-traumatic stress (IES≥35) reported at 1 week post-trauma were 10.2%, 12.3% and 13.5%, respectively, and showed a small decrease over time to 7.8%, 6.8% and 11.0% at 24 months post-trauma, respectively.

The results revealed that patients with symptoms of post-traumatic stress (IES≥35) showed worse outcomes on the EQ-5D-3L than patients with no symptoms of post-traumatic stress (Fig. 3).

-insert Fig3 here-

Prognostic factors of health status

Overall, HS measured as with the EQ-5D-3L increased over at least up until 6 months for all groups of patients and stabilized between 6 and 12 months post-trauma for most groups (see Table 3). Female patients had a lower HS compared to males at every time point.

At all time points, patients aged 85 and older had the lowest HS compared to the other age categories. At 3 and 6 months, all patient groups between 25 and 74 years reported the same HS

whereas patients aged between 18 and 24 reported a higher EQ-5D summary score. HS stabilized at 6 or 12 months for every age group, except for patients between 25 and 44 years for whom HS increased further.

Except for 1 week, patients with a high educational level had the highest HS. With an increasing number of comorbidities, HS decreased. Patients with moderate injuries (ISS 9-15) showed on almost each time point the lowest HS. At 1 week, severely injured patients (ISS ≥16) showed the lowest mean HS (0.28, SD 0.35).

Patients with the longest hospital LOS (≥15 days) had the lowest mean HS at all time points, ranging from 0.28 (SD 0.34) at 1 week up to 0.62 (SD 0.28) at 24 months after trauma.

Table 3. Mean (SD) of the self-reported health status for patient and injury characteristics as measured with the EQ-5D-3L.

		**Mean (SD) EQ-5D-3L summary score**						
**Characteristics**		**Pre-injury**	**1 week**	**1 month**	**3 months**	**6 months**	**12 months**	**24 months**
**Gender**	**Male**	0.90 (0.19)	0.54 (0.32)	0.62 (0.28)	0.74 (0.25)	0.79 (0.23)	0.83 (0.21)	0.84 (0.21)
	**Female**	0.80 (0.25)	0.43 (0.31)	0.50 (0.30)	0.64 (0.28)	0.69 (0.26)	0.70 (0.27)	0.72 (0.28)
**Age (yrs)**	**18-24**	0.95 (0,13)	0.50 (0.32)	0.63 (0.24)	0.79 (0.24)	0.85 (0.21)	0.86 (0.21)	0.86 (0.22)
	**25-44**	0.95 (0.12)	0.45 (0.30)	0.59 (0.29)	0.74 (0.24)	0.79 (0.24)	0.84 (0.22)	0.87 (0.19)
	**45-64**	0.91 (0.17)	0.49 (0.31)	0.60 (0.27)	0.73 (0.24)	0.79 (0.21)	0.83 (0.21)	0.83 (0.22)
	**65-74**	0.88 (0.20)	0.51 (0.31)	0.62 (0.28)	0.73 (0.24)	0.79 (0.22)	0.80 (0.22)	0.81 (0.23)
	**75-84**	0.78 (0.26)	0.52 (0.33)	0.53 (0.31)	0.66 (0.27)	0.70 (0.25)	0.70 (0.26)	0.70 (0.28)
	**≥85**	0.63 (0.28)	0.40 (0.33)	0.39 (0.32)	0.50 (0.29)	0.55 (0.29)	0.57 (0.29)	0.56 (0.30)
**Educational level**	**Low**	0.78 (0.27)	0.49 (0.33)	0.52 (0.31)	0.65 (0.28)	0.70 (0.27)	0.71 (0.27)	0.72 (0.28)
	**Middle**	0.90 (0.17)	0.50 (0.31)	0.59 (0.29)	0.71 (0.26)	0.77 (0.24)	0.80 (0.23)	0.81 (0.23)
	**High**	0.93 (0.13)	0.48 (0.30)	0.62 (0.26)	0.75 (0.23)	0.80 (0.21)	0.84 (0.20)	0.86 (0.20)
**Comorbidity**	**0**	0.96 (0.12)	0.53 (0.30)	0.65 (0.26)	0.77 (0.23)	0.83 (0.19)	0.86 (0.18)	0.87 (0.18)
	**1**	0.85 (0.21)	0.49 (0.32)	0.56 (0.29)	0.69 (0.25)	0.75 (0.24)	0.77 (0.24)	0.78 (0.25)
	**2**	0.75 (0.24)	0.45 (0.30)	0.47 (0.31)	0.61 (0.29)	0.66 (0.26)	0.67 (0.27)	0.68 (0.28)
	**≥3**	0.64 (0.29)	0.40 (0.33)	0.42 (0.32)	0.56 (0.29)	0.58 (0.29)	0.59 (0.29)	0.59 (0.29)
**ISS**	**1-3**	0.86 (0.22)	0.63 (0.29)	0.69 (0.27)	0.78 (0.23)	0.79 (0.24)	0.79 (0.25)	0.81 (0.25)
	**4-8**	0.89 (0.20)	0.46 (0.31)	0.56 (0.29)	0.71 (0.25)	0.77 (0.23)	0.80 (0.22)	0.81 (0.23)
	**9-15**	0.80 (0.26)	0.43 (0.30)	0.50 (0.30)	0.63 (0.28)	0.68 (0.27)	0.70 (0.28)	0.72 (0.28)
	**≥16**	0.90 (0.18)	0.37 (0.35)	0.50 (0.31)	0.65 (0.30)	0.74 (0.25)	0.77 (0.24)	0.77 (0.25)
**LOS (days)**	**≤2**	0.91 (0.18)	0.61 (0.29)	0.70 (0.25)	0.81 (0.21)	0.83 (0.20)	0.84 (0.22)	0.86 (0.21)
	**3-7**	0.85 (0.23)	0.47 (0.30)	0.55 (0.28)	0.69 (0.25)	0.74 (0.24)	0.77 (0.24)	0.79 (0.24)
	**8-14**	0.78 (0.27)	0.31 (0.31)	0.46 (0.29)	0.59 (0.29)	0.65 (0.27)	0.68 (0.28)	0.67 (0.29)
	**≥15**	0.74 (0.28)	0.28 (0.34)	0.32 (0.31)	0.50 (0.29)	0.58 (0.28)	0.60 (0.28)	0.62 (0.28)

Range of EQ-5D-3L: 0-1. Mean EQ-5D-3L of the general Dutch population: 0.87.
Abbreviations: SES, socio-economic status; SD, standard deviation; EQ-5D, EuroQol-5D-3L; ISS, Injury Severity Score; LOS, length of hospital stay; yrs, years

After adjustment for confounding factors, short-term (1 week and 1 month) prognostic factors for a significant lower EQ-5D summary score were female gender, a higher number of comorbidities, a longer LOS, a higher ISS, pelvic injury, tibia/complex foot or femur fracture, radius/ulna/hand fracture, shoulder/upper arm injury, rib fracture, spinal cord injury and stable vertebral fracture/disc injury (see Table 4). Mid-term (3 and 6 months) prognostic factors were a higher number of comorbidities, an ISS between 4 and 15, a longer LOS, radius/ulna/hand fracture, tibia/complex foot or femur fracture, severe TBI, spinal cord injury and

stable vertebral fracture/disc injury. Long-term (12 and 24 months) prognostic factors for a lower HS were: age 75 and above, 2 or more comorbidities, a longer LOS, tibia/complex foot or femur fracture, spinal cord injury and stable vertebral fracture/disc injury were prognostic factors. A high educational level was associated with higher HS in the long-term analysis.

Table 4. Multivariable longitudinal analysis of short, mid and long-term prognostic factors for decreased health status as measured with the EQ-5D-3L.

			**Total (1 week-24 months)**	**Short-term (1 week-1 month)**	**Mid-term (3-6 months)**	**Long-term (12-24 months)**
		**Number of patients**	*** Beta (95% C.I.)**	*** Beta (95% C.I.)**	*** Beta (95% C.I.)**	*** Beta (95% C.I.)**
**Characteristics**		**4,809**	**n=4,809**	**n=3,314**	**n=4,048**	**n=3,422**
**Gender**	Male	2,291	ref	ref	ref	ref
	Female	2,518	-0.05 (-0.06; -0.04)	-0.06 (-0.08; -0.05)	-0.05 (-0.06;-0.03)	-0.06 (-0.08; -0.05)
**Age (yrs)**	18-44	721	ref	ref	ref	ref
	45-64	1,345	0.04 (0.02; 0.05)	0.06 (0.04; 0.09)	0.02 (-0.00; 0.04)	0.01 (-0.01; 0.03)
	65-74	956	0.09 (0.08; 0.11)	0.14 (0.11; 0.17)	0.07 (0.05; 0.09)	0.04 (0.02; 0.06)
	≥75	1,787	0.02 (0.01; 0.04)	0.09 (0.06; 0.12)	-0.01 (-0.03; 0.01)	-0.05 (-0.07; -0.02)
**Educational level**	Low	2,637	ref	ref	ref	ref
	Middle	1,280	0.02 (0.00; 0.03)	0.01 (-0.01; 0.03)	0.01 (-0.00; 0.02)	0.03 (0.01; 0.04)
	High	893	0.03 (0.02; 0.05)	0.02 (-0,01; 0,04)	0.04 (0.02; 0.05)	0.05 (0.04; 0.07)
**Number of comor-bidities**	None	1,801	ref	ref	ref	ref
	1	1,426	-0.06 (-0.07; -0.05)	-0.06 (-0.08; -0.04)	-0.06 (-0.07; -0.04)	-0.05 (-0.07; -0.04)
	≥2	1,582	-0.15 (-0.16; -0.14)	-0.14 (-0.17; -0.12)	-0.14 (-0.16;-0.13)	-0.16 (-0.18; -0.15)
**ISS**	1-3	1,139	ref	ref	ref	ref
	4-8	1,596	0.00 (-0.01; 0.02)	-0.03 (-0.06; -0.01)	0.01 (-0.05; 0.03)	0.03 (0.01; 0.05)
	9-15	1,838	-0.02 (-0.04; -0.00)	-0.06 (-0.09; -0.02)	-0.02 (-0.04; -0.01)	0.00 (-0.02; 0.03)
	≥16	235	0.01 (-0.01; 0.04)	-0.02 (-0.08; 0.04)	0.01 (-0.04; 0.05)	0.04 (-0.01; 0.08)
**LOS**	≤2	1,425	ref	ref	ref	ref
	3-7	2,053	-0.05 (-0.06; -0.04)	-0.08 (-0.10; -0.06)	-0.06 (-0.07; -0.04)	-0.03 (-0.04; -0.01)
	8-14	975	-0.11 (-0.12; -0.09)	-0.15 (-0.18; -0.12)	-0.11 (-0.13; -0.09)	-0.08 (-0.11; -0.06)
	≥15	357	-0.20 (-0.22; -0.18)	-0.24 (-0.28; -0.20)	-0.20 (-0.23; -0.17)	-0.17 (-0.20; -0.14)
**Injury**	Pelvic injury	286	-0.05 (-0.07; -0.03)	-0.13 (-0.16; -0.10)	-0,01 (-0.04; 0.01)	0.00 (-0.02; 0.03)
	Hip fracture	1,242	-0.02 (-0.04; -0.01)	-0.02 (-0.05; 0.01)	-0.03 (-0.05; -0.00)	-0.02 (-0.05; 0.00)
	Tibia, complex foot or femur fracture	561	-0.05 (-0.06; -0.04)	-0.11 (-0.14; -0.08)	-0.05 (-0.07; -0.02)	-0.02 (-0.04; 0.00)
	Shoulder and upper arm injury	469	-0.03 (-0.04; -0.01)	-0.07 (-0.10; -0.05)	-0.02 (-0.04; 0.00)	-0.00 (-0.02; 0.02)
	Radius, ulna or hand fracture	304	0.00 (-0.02; 0.02)	-0.03 (-0.06; 0.00)	0.00 (-0.02; 0.03)	0.02 (-0.00; 0.05)
	Mild TBI (AIS 1-2)	1,302	0.03 (0.02; 0.04)	0.06 (0.04; 0.08)	0.03 (0.02; 0.05)	0.02 (-0.00; 0.03)
	Serious TBI (AIS 3)	125	0.04 (0.01; 0.07)	0.07 (0.02; 0.13)	0.05 (0.01; 0.10)	0.01 (-0.03; 0.05)
	Severe TBI (≥ 4)	76	-0.03 (-0.06; 0.02)	-0.02 (-0.10; 0.07)	-0.06 (-0.12; 0.00)	-0.01 (-0.07; 0.05)
	Facial fracture	243	0.02 (0.01; 0.04)	0.05 (0.01; 0.09)	0.02 (-0.01; 0.04)	0.01 (-0.01; 0.04)
	Thoracic injury	198	0.04 (0.02; 0.06)	0.05 (0.01; 0.10)	0.04 (0.02; 0.07)	0.03 (-0.01;

						0.06)
	Rib fracture	533	0.01 (-0.00; 0.03)	-0.01 (-0.04; 0.01)	0.03 (0.01; 0.05)	0.01 (-0.01; 0.04)
	Mild abdominal injury	85	0.02 (-0.02; 0.05)	0.04 (-0.02; 0.10)	0.01 (-0.04; 0.05)	0.02 (-0.03; 0.07)
	Severe abdominal injury	36	0.03 (-0.02; 0.08)	-0.05 (-0.15; 0.05)	0.06 (-0.04; 0.13)	0.05 (-0.02; 0.13)
	Spinal cord injury	27	-0.11 (-0.17; -0.05)	-0.10 (-0.22; 0.03)	-0.12 (-0.20; -0.03)	-0.15 (-0.23; -0.07)
	Stable vertebral fracture or disc injury	296	-0.05 (-0.07; -0.03)	-0.08 (-0.12; -0.05)	-0.05 (-0.08; -0.03)	-0.03 (-0.06; -0.01)

** Mixed models, adjusted for all other variables*
Beta: mean increase in EQ-5D-3L score (improvement of Health Status) compared to the reference category.
Range of EQ-5D-3L: 0-1. Mean EQ-5D-3L of the general Dutch population: 0.87 [47].
Abbreviations: ISS, Injury Severity Score; AIS, Abbreviated Injury Scale; LOS, Hospital lenght of stay in days; TBI, traumatic brain injury; yrs=years

Discussion

This study describes HS and psychological outcomes over 24 months after trauma. HS markedly improved during the 24 months after trauma, most of which occurred within the first 3 months. Compared to pre-injury HS, a large decrease in HS was found 1 week post-trauma. The prevalence rates of symptoms of anxiety and depression were relatively low. In contrast, symptoms of post-traumatic stress were highly prevalent and were present five times as often as compared with the Dutch general population [48]. Additionally, in line with the literature, having symptoms of post-traumatic stress were associated with a lower HS [27]. The addition of an assessment at 1 week post-trauma in the present study adds detailed insight into (baseline) functioning shortly after trauma. Therefore, it provides a more valid assessment of the magnitude of recovery thereafter.

Between 1 week and 3 months, the percentage of patients who reported problems on the ‘pain/discomfort’ and ‘self-care’ dimensions of the EQ-5D decreased steeply. For the ‘mobility’ and ‘usual activities’ dimensions, this decrease started 1 month after trauma. The percentage of patients who reported problems on the ‘anxiety and depression’ dimension was the highest at 1 month after trauma. Within 6 months post-trauma, patients showed the most recovery. From 6 months post-trauma onwards, little improvement in overall HS was found. The percentage of patients who reported improvements on the different EQ-5D domains increased through the 24 months of follow-up. However, the vast majority of trauma patients did not recover to their pre-injury HS. The mean EQ-5D-3L summary score for the total Dutch adult population is considered 0.87 (SD 0.18) (for males 0.89 (SD 0.16) and for females 0.85 (SD 0.19)) [47].

At short-term (up to 1 month post-trauma) female gender, lower extremity, spine, shoulder and upper arm injuries, injury severity, comorbidities and a longer hospital stay were associated with lower

HS. At mid-term (3 and 6 months), almost the same prognostic factors were significant and relevant, however, only injury severity seemed to be less important. In the long-term a greater age, two or more comorbidities, a longer hospital stay and only a few injuries (i.e., lower extremity fracture and spine injury) showed a significantly lower HS. The effect of injury severity seemed to fade over time. Spinal cord injury patients had the highest risk (long-term Beta -0,18, CI -0,27;-0,08) of a lower HS during both the short (not significant), mid and long-term post-trauma. Middle and high educational levels were associated with a higher HS in the long term compared to those with low educational levels.

Most recovery in HS occurs up to 3 months post-trauma, which is in agreement with previous studies on this topic [9, 10, 19]. In this regard, the addition of an assessment at 1 week post-trauma in the present study adds detailed insight into (baseline) functioning shortly after trauma. Prior work also confirms the finding that a large proportion of patients have a considerably lower HS 1 year post-trauma compared to pre-injury HS [9, 11, 16] or compared to the HS of the general population [49].

In addition to the physical injury itself, other characteristics largely affect HS after trauma. This was particularly in the long-term. This finding extends those of previous studies [15, 35], confirming that patients who were the most severely injured, were not necessarily those with the lowest HS.

In this study, the prevalence of symptoms of anxiety and depression was slightly higher compared to the prevalence of an anxiety disorder or depression in the general Dutch population (both disorders are estimated to be present in 10.0% of the Dutch population) [50, 51]. As a result, the prevalence of symptoms of anxiety and depression slightly decreased over time. Recent and comparable studies have documented a slightly higher prevalences of symptoms of anxiety and depression [9, 16].

The prevalence of symptoms of post-traumatic stress was high as compared with the Dutch population (11.0% at 2 years post-trauma versus 2.6-3.3%) [48]. Our prevalence rate of long-term symptoms of post-traumatic stress is in line with previous research that also uses the ≥35 cut-off point for the IES [13]. However, the systematic review by Haagsma *et al.* [52] revealed prevalence of post-traumatic stress in hospitalized trauma patients that ranged from 30% (90% CI 27%-33%) within 3 months post-trauma to 6% (90% CI 4%-10%) at 1 year. Compared to those results, we found a lower prevalence of symptoms of post-traumatic stress early post-trauma whereas a higher prevalence was found at the 1 year follow-up. This discrepancy may be due to studies using various instruments and different cut-off points to indicate post-traumatic stress.

This study was conducted according to the recommended guidelines for measuring non-fatal outcomes after trauma [41]. The BIOS included a broad study population, measured both short-term and long-term outcomes, measured functioning prior to the trauma, included a large number of patient and injury-related characteristics and requested information from proxy informants for patients who were incapable of completing the set of questionnaires themselves. Recruitment for the BIOS occurred in all hospitals of the Dutch Noord-Brabant region, covering both urban and rural populations.

The inclusion of elderly individuals with a hip fracture in the current study improves the generalizability of the study findings. In this study, one out of four participants was aged 65 or older and had a hip fracture. We are aware that recovery patterns (due to comorbidities and functional decline) in the elderly population are different from recovery patterns in younger patients.

This study also has several limitations. First, there was selection bias since younger patients, elderly patients, patients with very minor injuries (ISS 1-3) and those with a low status score (used as a proxy to indicate SES; a low status score indicate a lower SES-level) were less likely to participate. It is challenging to include these specific groups. Previous studies also reported lower response rates from younger and elderly patients [7, 10, 24, 35], from patients with minor injuries [10] and from those with low educational level [53, 54]. Second, only a selected group of patients (18% of the eligible population) completed the 1 week assessment. Since most recovery occurs within the first 3 months post-trauma, it is vital to examine very early recovery patterns. This study provides unique data since it incorporates the use of a standardized 1 week assessment in a comprehensive group of trauma patients. Nevertheless in most cases, non-responders at this time point felt too disabled to complete a long questionnaire. Therefore, this most likely led to an underestimations of the reported HS and psychological outcomes 1 week post-trauma. Third, there were many missing data, especially early post-trauma. However, we were able to impute missing sum scores in several cases. Fourth, we did not correct for pre-injury HS in the longitudinal analyses since we were not able to collect pre-injury HS in all participants. Fifth, we did not include a pre-injury assessment of the symptoms of anxiety or depression. Sixth, we cannot exclude change findings due to multiple testing. Change findings might have occurred for the results of the longitudinal analyses as presented in Table 4.

Given the acute nature of trauma, it is difficult to include patients soon after they experience trauma in order to examine very early recovery patterns. To increase the response rate and to reduce loss to follow-up, future research should minimize the large number of questionnaires that patients

have to complete at each time point, especially early post-trauma. A promising technique includes computerized adaptive testing. In this already proven valid technique, tailored-made short and precise domain-specific data can be collected [55-58].

We are aware that recall bias and response shift most likely led to an overestimation of the pre-injury HS as measured in this study. However, to produce valid estimates of the health impact and the decrease in functioning after trauma, information on patients functioning prior to the trauma is crucial [59-62]. Future research should focus on the effects of recall bias and response shift on retrospectively collected data.

Implications for health-care

According to the literature, early recognition, treatment and monitoring of psychological problems improve non-fatal outcomes after trauma [16, 63-66]. Therefore, early screening and interventions to reduce symptoms of post-traumatic stress should be part of standard care. Furthermore in the long long-term, patients aged ≥75 years, patients with a longer length of hospital stay and patients with ≥2 comorbidities are more likely to have a poor HS. For these patients, standard aftercare should be extended to screen for remaining problems that the patients should address after their trauma. For example by a follow-up appointment with a case manager.

Symptoms of post-traumatic stress were frequently reported in this study. Patients need to be better informed about the psychological problems they may experience after trauma. Health-care providers should not solely focus on the physical consequences but should also be aware of all other possible consequences that may occur after trauma. To achieve this, a more holistic approach towards the treatment of trauma patients should be aimed in which the patients’ own perspectives on their recovery should play a crucial role.

Conclusion

Hospitalized trauma patients experience substantial reductions in HS and frequently report symptoms of post-traumatic stress. The results of this study should be interpreted with caution since younger patients, patients with very minor injuries and those with a lower status score (used as an proxy to indicate SES, a lower status score indicates a lower SES-level) were less likely to participate.

Most improvements in HS and psychological symptoms occurred within the first 3 months post-trauma. After two years post trauma, the vast majority of trauma patients did not achieve their pre-injury HS. Recovery trajectories varied widely in which female gender, age ≥75 years, spinal cord injury, having more comorbidities, low educational level, a longer hospital stay and symptoms of post-traumatic stress were associated with a higher risk of decreased HS in the long-term after trauma. In the short-term, also several lower extremity injuries are prognostic factors for decreased HS.

Acknowledgements

Memberschip list of the BIOS-group: P.V. van Eerten (Maxima Medical Center, department of surgery, Eindhoven, the Netherlands), F.C. van Eijck (Bravis Hospital, department of surgery, Bergen op Zoom and Roosendaal, the Netherlands), H.J. van Geffen (Jeroen Bosch Hospital, department of surgery, ‘s-Hertogenbosch, the Netherlands), W.A. Haagh (St. Anna Hospital, department of surgery, Geldrop, the Netherlands), L.M. Poelhekke (Maasziekenhuis Pantein Hospital, department of surgery, Boxmeer, the Netherlands), J.B. Sintenie (Elkerliek Hospital, department of surgery, Helmond, the Netherlands, C.T. Stevens (Bernhoven Hospital, department of surgery, Uden, the Netherlands), A.H. van der Veen (Catharina Hospital, department of surgery, Eindhoven, the Netherlands), C.H. van der Vlies (Maasstad Hospital, department of surgery, Rotterdam, Eindhoven, D.I. Vos (Amphia Hospital, department of surgery, Breda, the Netherlands). Contact person of the BIOS-group: A.H. van der Veen: alexander.vd.veen@catharinaziekenhuis.nl

References

1. Murray CJ, Vos T, Lozano R, Naghavi M, Flaxman AD, Michaud C, et al. Disability-adjusted life years (DALYs) for 291 diseases and injuries in 21 regions, 1990-2010: a systematic analysis for the Global Burden of Disease Study 2010. Lancet. 2012;380(9859):2197-223. doi: 10.1016/S0140-6736(12)61689-4. PubMed PMID: 23245608.
2. WorldHealthOrganization. Injuries and violence, the facts. 2014.
3. Haider AH, Herrera-Escobar JP, Al Rafai SS, Harlow AF, Apoj M, Nehra D, et al. Factors Associated With Long-Term Outcomes After Injury: Results of the Functional Outcomes and Recovery After Trauma Emergencies (FORTE) Multicenter Cohort Study. Ann Surg. 2018. Epub 2018/12/15. doi: 10.1097/SLA.0000000000003101. PubMed PMID: 30550382.
4. Lyons RA, Kendrick D, Towner EM, Christie N, Macey S, Coupland C, et al. Measuring the population burden of injuries--implications for global and national estimates: a multi-centre prospective UK longitudinal study. PLoS Med. 2011;8(12):e1001140. doi: 10.1371/journal.pmed.1001140. PubMed PMID: 22162954; PubMed Central PMCID: PMCPMC3232198.
5. Bhalla K, Harrison J, Abraham J, Borse NN, Lyons R, Boufous S, et al. Data sources for improving estimates of the global burden of injuries: call for contributors. PLoS Med. 2009;6(1):e1. doi: 10.1371/journal.pmed.1000001. PubMed PMID: 19166263; PubMed Central PMCID: PMCPMC2628399.
6. Polinder S, Haagsma JA, Lyons RA, Gabbe BJ, Ameratunga S, Cryer C, et al. Measuring the population burden of fatal and nonfatal injury. Epidemiol Rev. 2012;34:17-31. doi: 10.1093/epirev/mxr022. PubMed PMID: 22113244.
7. Gabbe BJ, Simpson PM, Cameron PA, Ponsford J, Lyons RA, Collie A, et al. Long-term health status and trajectories of seriously injured patients: A population-based longitudinal study. PLoS Med. 2017;14(7):e1002322. doi: 10.1371/journal.pmed.1002322. PubMed PMID: 28678814; PubMed Central PMCID: PMCPMC5497942.
8. de Vries J. Quality of life assessment, in Assessment in behavorial medicine, A.J.J.M. Vingerhoets, Editor 2001, Brunner-Routledge: Hove. 2001.

9. Kendrick D, Kelllezi B, Coupland C, Maula A, Beckett K, Morriss R, et al. Psychological morbidity and health-related quality of life after injury: multicentre cohort study. Qual Life Res. 2017;26(5):1233-50. doi: 10.1007/s11136-016-1439-7. PubMed PMID: 27785608; PubMed Central PMCID: PMCPMC5376395.

10. Meerding WJ, Looman CW, Essink-Bot ML, Toet H, Mulder S, van Beeck EF. Distribution and determinants of health and work status in a comprehensive population of injury patients. J Trauma. 2004;56(1):150-61. doi: 10.1097/01.TA.0000062969.65847.8B. PubMed PMID: 14749582.

11. Harcombe H, Langley J, Davie G, Derrett S. Functional status following injury: what recovery pathways do people follow? Injury. 2015;46(7):1275-80. doi: 10.1016/j.injury.2015.04.004. PubMed PMID: 25920372.

12. Polinder S, van Beeck EF, Essink-Bot ML, Toet H, Looman CW, Mulder S, et al. Functional outcome at 2.5, 5, 9, and 24 months after injury in the Netherlands. J Trauma. 2007;62(1):133-41. doi: 10.1097/TA.0b013e31802b71c9. PubMed PMID: 17215744.

13. van Delft-Schreurs CC, van Bergen JJ, van de Sande P, Verhofstad MH, de Vries J, de Jongh MA. A cross-sectional study of psychological complaints and quality of life in severely injured patients. Qual Life Res. 2014;23(4):1353-62. doi: 10.1007/s11136-013-0546-y. PubMed PMID: 24202637.

14. Haagsma JA, Polinder S, Olff M, Toet H, Bonsel GJ, van Beeck EF. Posttraumatic stress symptoms and health-related quality of life: a two year follow up study of injury treated at the emergency department. BMC Psychiatry. 2012;12:1. doi: 10.1186/1471-244X-12-1. PubMed PMID: 22230388; PubMed Central PMCID: PMCPMC3276433.

15. van Delft-Schreurs CC, van Bergen JJ, de Jongh MA, van de Sande P, Verhofstad MH, de Vries J. Quality of life in severely injured patients depends on psychosocial factors rather than on severity or type of injury. Injury. 2014;45(1):320-6. doi: 10.1016/j.injury.2013.02.025. PubMed PMID: 23510753.

16. Kellezi B, Coupland C, Morriss R, Beckett K, Joseph S, Barnes J, et al. The impact of psychological factors on recovery from injury: a multicentre cohort study. Soc Psychiatry Psychiatr Epidemiol. 2017;52(7):855-66. doi: 10.1007/s00127-016-1299-z. PubMed PMID: 27803978; PubMed Central PMCID: PMCPMC5504249.

17. Skogstad L, Toien K, Hem E, Ranhoff AH, Sandvik L, Ekeberg O. Psychological distress after physical injury: a one-year follow-up study of conscious hospitalised patients. Injury. 2014;45(1):289-98. doi: 10.1016/j.injury.2012.10.001. PubMed PMID: 23103082.

18. Wiseman TA, Curtis K, Lam M, Foster K. Incidence of depression, anxiety and stress following traumatic injury: a longitudinal study. Scand J Trauma Resusc Emerg Med. 2015;23:29. doi: 10.1186/s13049-015-0109-z. PubMed PMID: 25882818; PubMed Central PMCID: PMCPMC4389309.

19. Kendrick D, Vinogradova Y, Coupland C, Mulvaney CA, Christie N, Lyons RA, et al. Recovery from injury: the UK burden of injury multicentre longitudinal study. Inj Prev. 2013;19(6):370-81. doi: 10.1136/injuryprev-2012-040658. PubMed PMID: 23599210.

20. Langley J, Derrett S, Davie G, Ameratunga S, Wyeth E. A cohort study of short-term functional outcomes following injury: the role of pre-injury socio-demographic and health characteristics, injury and injury-related healthcare. Health Qual Life Outcomes. 2011;9:68. doi: 10.1186/1477-7525-9-68. PubMed PMID: 21849082; PubMed Central PMCID: PMCPMC3170567.

21. Derrett S, Davie G, Ameratunga S, Wyeth E, Colhoun S, Wilson S, et al. Prospective Outcomes of Injury Study: recruitment, and participant characteristics, health and disability status. Inj Prev. 2011;17(6):415-8. doi: 10.1136/injuryprev-2011-040044. PubMed PMID: 21724742.

22. Mackenzie EJ, Rivara FP, Jurkovich GJ, Nathens AB, Frey KP, Egleston BL, et al. The National Study on Costs and Outcomes of Trauma. J Trauma. 2007;63(6 Suppl):S54-67; discussion S81-6. doi: 10.1097/TA.0b013e31815acb09. PubMed PMID: 18091213.

23. Lyons RA, Towner EE, Kendrick D, Christie N, Brophy S, Phillips CJ, et al. The UK burden of injury study - a protocol. [National Research Register number: M0044160889]. BMC Public Health. 2007;7:317. doi: 10.1186/1471-2458-7-317. PubMed PMID: 17996057; PubMed Central PMCID: PMCPMC2225415.

24. Langley J, Davie G, Wilson S, Lilley R, Ameratunga S, Wyeth E, et al. Difficulties in functioning 1 year after injury: the role of preinjury sociodemographic and health characteristics, health care and injury-related factors. Arch Phys Med Rehabil. 2013;94(7):1277-86. doi: 10.1016/j.apmr.2013.02.012. PubMed PMID: 23439409.

25. Gabbe BJ, Simpson PM, Harrison JE, Lyons RA, Ameratunga S, Ponsford J, et al. Return to Work and Functional Outcomes After Major Trauma: Who Recovers, When, and How Well? Ann Surg. 2016;263(4):623-32. doi: 10.1097/SLA.0000000000001564. PubMed PMID: 26779977.

26. Gabbe BJ, Simpson PM, Lyons RA, Ameratunga S, Harrison JE, Derrett S, et al. Association between the number of injuries sustained and 12-month disability outcomes: evidence from the injury-VIBES study. PLoS One. 2014;9(12):e113467. doi: 10.1371/journal.pone.0113467. PubMed PMID: 25501651; PubMed Central PMCID: PMCPMC4263479.

27. Giummarra MJ, Casey SL, Devlin A, Ioannou LJ, Gibson SJ, Georgiou-Karistianis N, et al. Co-occurrence of posttraumatic stress symptoms, pain, and disability 12 months after traumatic injury. Pain Rep. 2017;2(5):e622. doi: 10.1097/PR9.0000000000000622. PubMed PMID: 29392235; PubMed Central PMCID: PMCPMC5777683.

28. de Jongh MA, Kruithof N, Gosens T, van de Ree CL, de Munter L, Brouwers L, et al. Prevalence, recovery patterns and predictors of quality of life and costs after non-fatal injury: the Brabant Injury Outcome Surveillance (BIOS) study. Inj Prev. 2016. doi: 10.1136/injuryprev-2016-042032. PubMed PMID: 27154507.

29. Linn BS, Linn MW, Gurel L. Cumulative illness rating scale. J Am Geriatr Soc. 1968;16(5):622-6. PubMed PMID: 5646906.

30. EuroQol G. EuroQol--a new facility for the measurement of health-related quality of life. Health Policy. 1990;16(3):199-208. PubMed PMID: 10109801.

31. Furlong W, Feeny D, Torrance G. Health Utilities Index (HUI) procedures manual: algorithm for determining HUI Mark 2 (HUI2)/Mark 3 (HUI3) health status classification levels, health states single-attribute level utility scores and overall health-related quality of life utility scores from 15-item self-complete health status questionnaires. 2000.

32. Zigmond AS, Snaith RP. The hospital anxiety and depression scale. Acta Psychiatr Scand. 1983;67(6):361-70. PubMed PMID: 6880820.

33. Wohlfarth TD, van den Brink W, Winkel FW, ter Smitten M. Screening for Posttraumatic Stress Disorder: an evaluation of two self-report scales among crime victims. Psychol Assess. 2003;15(1):101-9. PubMed PMID: 12674729.

34. Kendrick D, Vinogradova Y, Coupland C, Christie N, Lyons RA, Towner EL, et al. Getting back to work after injury: the UK Burden of Injury multicentre longitudinal study. BMC Public Health. 2012;12:584. doi: 10.1186/1471-2458-12-584. PubMed PMID: 22853715; PubMed Central PMCID: PMCPMC3444403.

35. Ahmed W, Alwe R, Wade D. One-year functional outcomes following major trauma: experience of a UK level 1 major trauma centre. Clin Rehabil. 2017;31(12):1646-52. doi: 10.1177/0269215517712044. PubMed PMID: 28580790.

36. Polinder S, Haagsma JA, Belt E, Lyons RA, Erasmus V, Lund J, et al. A systematic review of studies measuring health-related quality of life of general injury populations. BMC Public Health. 2010;10:783. doi: 10.1186/1471-2458-10-783. PubMed PMID: 21182775; PubMed Central PMCID: PMCPMC3019196.

37. Polinder S, Haagsma JA, Bonsel G, Essink-Bot ML, Toet H, van Beeck EF. The measurement of long-term health-related quality of life after injury: comparison of EQ-5D and the health utilities index. Inj Prev. 2010;16(3):147-53. doi: 10.1136/ip.2009.022418. PubMed PMID: 20570982.

38. Manuel DG, Schultz SE, Kopec JA. Measuring the health burden of chronic disease and injury using health adjusted life expectancy and the Health Utilities Index. J Epidemiol Community Health. 2002;56(11):843-50. PubMed PMID: 12388577; PubMed Central PMCID: PMCPMC1732044.

39. Nguyen H, Ivers R, Pham C. Health-related quality of life and recovery patterns among hospitalised injury patients in Vietnam. Qual Life Res. 2018;27(3):619-29. doi: 10.1007/s11136-017-1724-0. PubMed PMID: 29079888.

40. Gabbe BJ, Lyons RA, Sutherland AM, Hart MJ, Cameron PA. Level of agreement between patient and proxy responses to the EQ-5D health questionnaire 12 months after injury. J Trauma Acute Care Surg. 2012;72(4):1102-5.

41. Van Beeck EF, Larsen CF, Lyons RA, Meerding WJ, Mulder S, Essink-Bot ML. Guidelines for the conduction of follow-up studies measuring injury-related disability. J Trauma. 2007;62(2):534-50. doi: 10.1097/TA.0b013e31802e70c7. PubMed PMID: 17297349.

42. Lamers LM, McDonnell J, Stalmeier PF, Krabbe PF, Busschbach JJ. The dutch tariff: Results and arguments for an effective design for national EQ-5D valuation studies. Health Econ 2006;15(10):1121-32.

43. Committee on Injury Scaling, Association for the Advancement of Automotive Medicine (AAAM). The Abbreviated Injury Scale, 1990 Revision. 2008.

44. SociaalenCultureelPlanbureau. Veelgestelde vragen over statusscore, versie 5 maart 2015.

45. van Buuren S, Oudshoorn K. Flexible multivariate imputation by MICE. Leiden: Netherlands Organization for Applied Scientific Research (TNO) Prevention and Health, 1999.

46. Laird NM, Ware JH. Random-effects models for longitudinal data. Biometrics. 1982;38(4):963-74. PubMed PMID: 7168798.

47. Stolk E, Krabbe P, Busschbach J. Using the Internet to collect EQ-5D norm scores: a valid alternative? 24th Scientific Plenary Meeting of the EuroQoL Group - Proceedings: 153-1652009.

48. . Available from: https://www.volksgezondheidenzorg.info/onderwerp/posttraumatische-stressstoornis/cijfers-context/huidige-situatie#node-prevalentie-van-ptss-de-bevolking.

49. Toien K, Bredal IS, Skogstad L, Myhren H, Ekeberg O. Health related quality of life in trauma patients. Data from a one-year follow up study compared with the general population. Scand J Trauma Resusc Emerg Med. 2011;19:22. doi: 10.1186/1757-7241-19-22. PubMed PMID: 21477280; PubMed Central PMCID: PMCPMC3088534.

50. Verweij G, Houben-van Herten M. Depressiviteit en antidepressiva in Nederland, bevolkingstrends 2013. CBS, December 2013.

51. de Graaf R, ten Have M, van Dorsselaer S. De psychische gezondheid van de Nederlandse bevolking. NEMESIS-2: Opzet en eerste resultaten. Utrecht: Trimbos-insituut. Netherlands Institute of Mental Health and Addiction, 2010.

52. Haagsma JA, Polinder S, Toet H, Panneman M, Havelaar AH, Bonsel GJ, et al. Beyond the neglect of psychological consequences: post-traumatic stress disorder increases the non-fatal burden of injury by more than 50%. Inj Prev. 2011;17(1):21-6. doi: 10.1136/ip.2010.026419. PubMed PMID: 20929853.

53. Zarzaur BL, Bell T. Trajectory subtypes after injury and patient-centered outcomes. J Surg Res. 2016;202(1):103-10. doi: 10.1016/j.jss.2015.12.038. PubMed PMID: 27083954; PubMed Central PMCID: PMCPMC4834145.

54. Soberg HL, Bautz-Holter E, Finset A, Roise O, Andelic N. Physical and mental health 10 years after multiple trauma: A prospective cohort study. J Trauma Acute Care Surg. 2015;78(3):628-33. doi: 10.1097/TA.0000000000000541. PubMed PMID: 25710437.

55. Revicki DA, Cella DF. Health status assessment for the twenty-first century: item response theory, item banking and computer adaptive testing. Qual Life Res. 1997;6(6):595-600. PubMed PMID: 9330558.

56. Hung M, Stuart AR, Higgins TF, Saltzman CL, Kubiak EN. Computerized Adaptive Testing Using the PROMIS Physical Function Item Bank Reduces Test Burden With Less Ceiling Effects Compared With the Short Musculoskeletal Function Assessment in Orthopaedic Trauma Patients. J Orthop Trauma. 2014;28(8):439-43. doi: 10.1097/BOT.0000000000000059. PubMed PMID: 24378399.

57. Hung M, Franklin JD, Hon SD, Cheng C, Conrad J, Saltzman CL. Time for a paradigm shift with computerized adaptive testing of general physical function outcomes measurements. Foot Ankle Int. 2014;35(1):1-7. doi: 10.1177/1071100713507905. PubMed PMID: 24101733.

58. Jayakumar P, Overbeek C, Vranceanu AM, Williams M, Lamb S, Ring D, et al. The use of computer adaptive tests in outcome assessments following upper limb trauma. Bone Joint J. 2018;100-B(6):693-702. doi: 10.1302/0301-620X.100B6.BJJ-2017-1349.R1. PubMed PMID: 29855231.

59. Cubbin C, Smith GS. Socioeconomic inequalities in injury: critical issues in design and analysis. Annu Rev Public Health. 2002;23:349-75. doi: 10.1146/annurev.publhealth.23.100901.140548. PubMed PMID: 11910067.

60. Haagsma JA, Maertens de Noordhout C, Polinder S, Vos T, Havelaar AH, Cassini A, et al. Assessing disability weights based on the responses of 30,660 people from four European countries. Popul Health Metr. 2015;13:10. doi: 10.1186/s12963-015-0042-4. PubMed PMID: 26778920; PubMed Central PMCID: PMCPMC4715333.

61. MacKenzie EJ, Siegel JH, Shapiro S, Moody M, Smith RT. Functional recovery and medical costs of trauma: an analysis by type and severity of injury. J Trauma. 1988;28(3):281-97. PubMed PMID: 3351987.

62. Holtslag HR, van Beeck EF, Lindeman E, Leenen LP. Determinants of long-term functional consequences after major trauma. J Trauma. 2007;62(4):919-27. doi: 10.1097/01.ta.0000224124.47646.62. PubMed PMID: 17426549.

63. Kendrick D, Dhiman P, Kellezi B, Coupland C, Whitehead J, Beckett K, et al. Psychological morbidity and return to work after injury: multicentre cohort study. Br J Gen Pract. 2017;67(661):e555-e64. doi: 10.3399/bjgp17X691673. PubMed PMID: 28630058; PubMed Central PMCID: PMCPMC5519127.

64. Wilson SJ, Davie G, Derrett S. Two years after injury: prevalence and early post-injury predictors of ongoing injury-related problems. Qual Life Res. 2017;26(7):1831-8. doi: 10.1007/s11136-017-1521-9. PubMed PMID: 28229327.

65. Richmond TS, Kauder D, Hinkle J, Shults J. Early predictors of long-term disability after injury. Am J Crit Care. 2003;12(3):197-205. PubMed PMID: 12751393.

66. Jacoby SF, Shults J, Richmond TS. The effect of early psychological symptom severity on long-term functional recovery: A secondary analysis of data from a cohort study of minor injury patients. Int J Nurs Stud. 2017;65:54-61. doi: 10.1016/j.ijnurstu.2016.11.004. PubMed PMID: 27863295; PubMed Central PMCID: PMCPMC5148666.

67. Haagsma JA, Graetz N, Bolliger I, Naghavi M, Higashi H, Mullany EC, et al. The global burden of injury: incidence, mortality, disability-adjusted life years and time trends from the Global Burden of Disease study 2013. Inj Prev. 2016;22(1):3-18. doi: 10.1136/injuryprev-2015-041616. PubMed PMID: 26635210; PubMed Central PMCID: PMCPMC4752630.

Compliance with ethical standards

Ethical approval and informed consent: The BIOS was approved by the Medical Ethics Committee Brabant, the Netherlands (project number NL50258.028.14 and NW2016-09). This study was conducted according to the 1964 Declaration of Helsinki and its later amendements. Informed consent was obtained from all individual participants included in the study.

Availability of data and material: Data of the BIOS are unsuitable for public deposition due to the privacy of participant data. Data are anonymized, but due to relatively few severe cases, patients could be identified. Therefore, BIOS data are available for any interested researcher who meets the criteria for access to confidential data. The Brabant Trauma Registry (e-mail: secretariaat@nazb.nl) may be contacted to request data.

Competing interests: The authors declare that there is no conflict of interest.

Author's contributions:

NK: conception and design, acquisition of data, analysis and interpretation of data, drafting the article, critical revision of the article, final approval of the version to be published

SP: conception and design, analysis and interpretation of data, drafting the article, critical revision of the article, final approval of the version to be published

LM: conception and design, acquisition of data, analysis and interpretation of data, drafting the article, critical revision of the article, final approval of the version to be published

CR: conception and design, acquisition of data, analysis and interpretation of data, critical revision of the article, final approval of the version to be published

KL: conception and design, analysis and interpretation of data, final approval of the version to be published

MJ: conception and design, analysis and interpretation of data, drafting the article, critical revision of the article, final approval of the version to be published

BIOS-group: conception and design, final approval of the version to be published

Supporting information

S1 Table. Injury group classification of the most common types of injury, based on the Abbreviated Injury Score [67].

S2 File. Methods of imputed data of the Brabant Injury Outcome Surveillance.

S3 Table. Missing sum scores of the original data and imputed data of the self-reported health status and psychological measures of the participants of the Brabant Injury Outcome Surveillance (n=4,883).

729

730

Figure 1

Click here to access/download;Figure;Fig1_29122019.tiff

Fig 1. Number of participants throughout the Brabant Injury Outcome Surveillance (n=4,883).
Abbreviations: EQ-5D, EuroQol-5D-3L; HUI, Health Utilities Index Mark 2/3; HADS, Hospital Anxiety and Depression Scale; IES, Impact of Event Scale; VAS, Visual Analogue Scale.

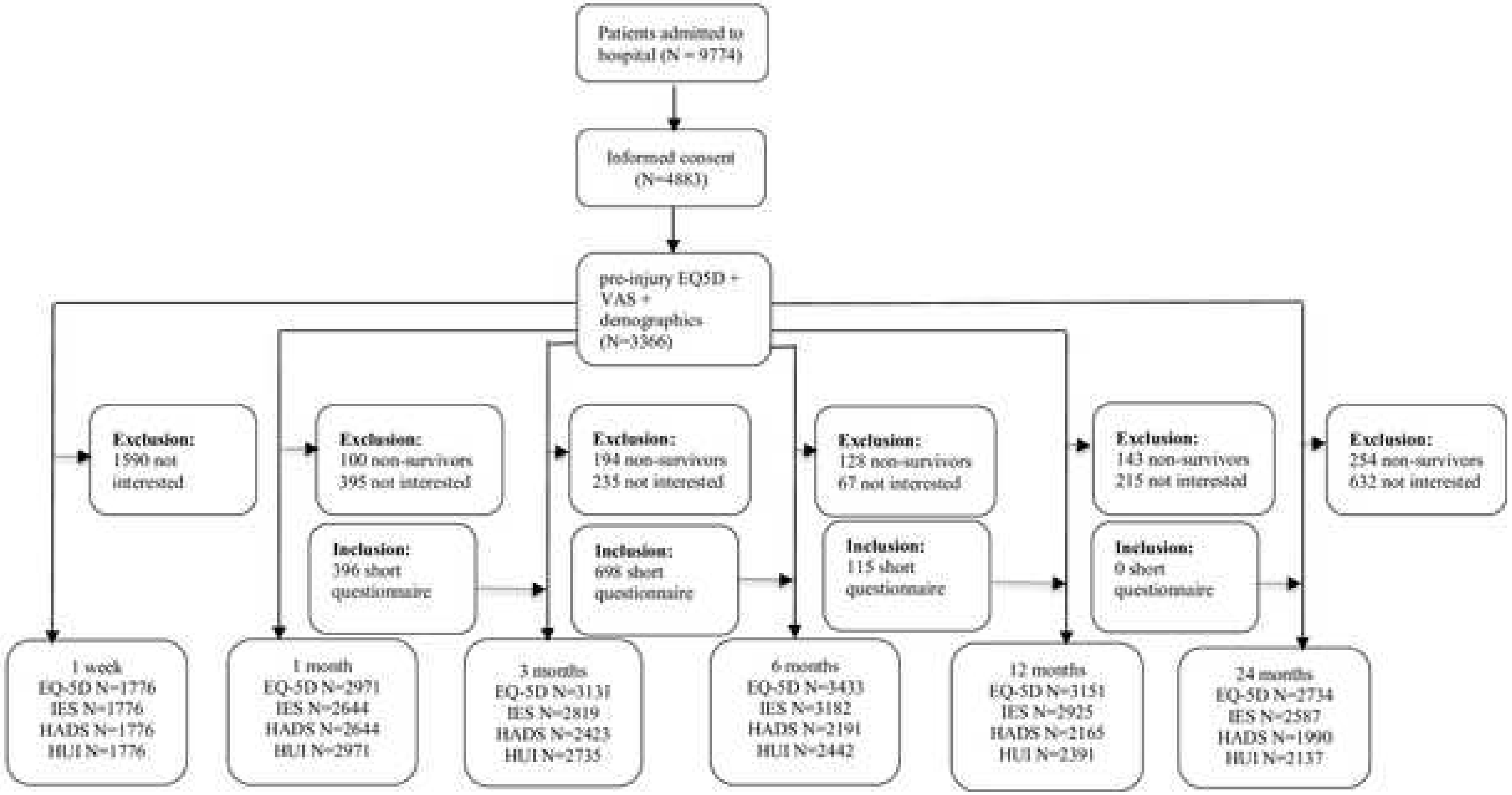

 Click here to access/download;Figure;Fig2_15112019.tiff

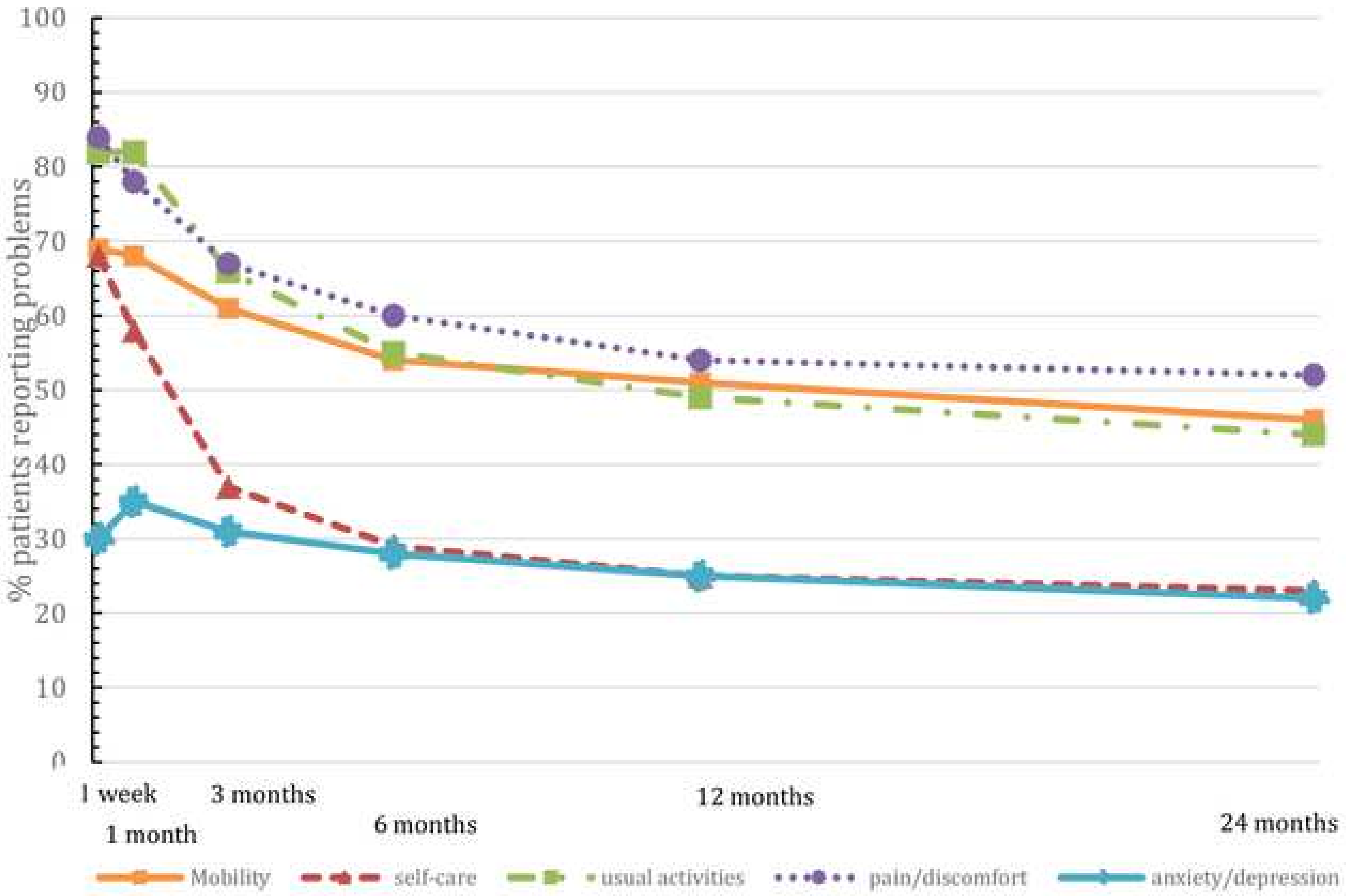

Fig 21. Prevalence of moderate or severe problems (%) on each EuroQol-5D-3L dimension up until 2 years of follow-up.

Figure 3

Click here to access/download;Figure;Figure 3_hersubmission v 2_20032020 kopie.tiff

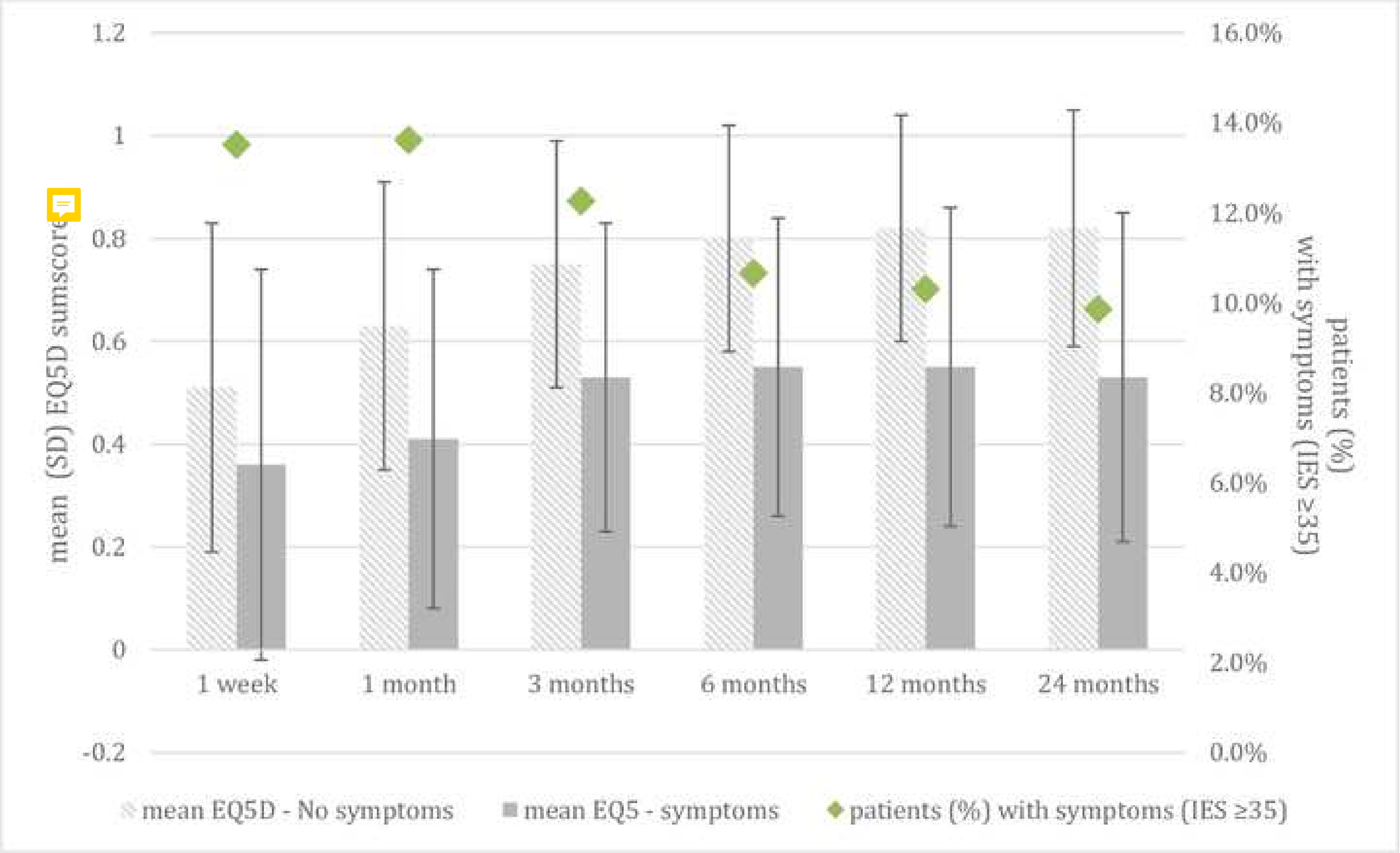

Click here to access/download
Supporting Information
S2_File_10112019.docx

Click here to access/download
Supporting Information
S3_Table_30122019.docx

Click here to access/download
Supporting Information
S2_File_10112019 clean.docx

Click here to access/download
Supporting Information
S3_Table_30122019 clean.docx

Click here to access/download
Supporting Information
S1_Table_10112019.docx

Health status and psychological outcomes after trauma; a prospective multicenter cohort study

Nena Kruithof[1*], Suzanne Polinder[2], Leonie de Munter[1], Cornelis L.P. van de Ree[1], Koen W.W. Lansink[1,3,4], Mariska A.C. de Jongh[1,4], BIOS-group

[1]ETZ Hospital (Elisabeth-TweeSteden Ziekenhuis), Department Trauma TopCare, Tilburg, the Netherlands

[2]Erasmus University Medical Centre, Department of Public Health, Rotterdam, the Netherlands

[3]ETZ Hospital (Elisabeth-TweeSteden Ziekenhuis), Department of Surgery, Tilburg, the Netherlands

[4]Brabant Trauma Registry, Network Emergency Care Brabant, Tilburg, the Netherlands

***Corresponding author:**

Email: nenakruithof@hotmail.com

Membership of the BIOS group is provided in the Acknowledgements

Abstract

Introduction: Survival after trauma has considerably improved. This warrants research on non-fatal outcome. We aimed to ~~describe the recovery patterns of health status (HS) and psychological outcomes during 24 months of follow-up and to~~ identify subgroups at risk of both short and long-term health status (HS) after trauma and to describe the recovery patterns of HS and psychological outcomes during 24 months of follow-up. **Methods:** Hospitalized patients with all types of injuries were included. Data were collected at 1 week, 1, 3, 6, 12, and 24 months post-trauma. HS was assessed with the EuroQol-5D-3L (EQ-5D-3L) and the Health Utilities Index Mark 2 and 3 (HUI2/3). For the screening of symptoms of post-traumatic stress, anxiety and depression, the Impact of Event Scale (IES) and the Hospital Anxiety and Depression Scale (HADS) subscale anxiety (HADSA) and subscale depression (HADSD) were used. Recovery patterns of HS and psychological outcomes were examined with linear mixed model analyses. **Results:** A total of 4,883 patients participated (median age 68 (Interquartile range 53-80); 50% response rate). The mean (Standard Deviation (SD)) pre-injury EQ-5D-3L score was 0.85 (0.23). One week post-trauma, mean (SD) EQ-5D-3L, HUI2 and HUI3 scores were 0.49 (0.32), 0.61 (0.22) and 0.38 (0.31), respectively. These scores significantly improved to 0.77 (0.26), 0.77 (0.21) and 0.62 (0.35), respectively, at 24 months. Most recovery occurred up until 3 months. At long-term follow-up, patients of higher age, with comorbidities, longer hospital stay, lower extremity fracture and spine injury showed lower HS. The mean (SD) scores of the IES, HADSA and HADSD were respectively 14.80 (15.80), 4.92 (3.98) and 5.00 (4.28), respectively, at 1 week post-trauma and slightly improved over 24 months post-trauma to 10.35 (14.72), 4.31 (3.76) and 3.62 (3.87), respectively. **Discussion:** HS and psychological symptoms improved over time and most improvements occurred within 3 months post-trauma. The effects of severity and type of injury faded out over time. Patients frequently reported symptoms of post-traumatic stress.

Trail registration: ClinicalTrials.gov identifier: NCT02508675

Keywords: injury, trauma, hospitalization, longitudinal cohort study, health status, psychological outcome, determinants, prognostic factors

Introduction

Trauma poses a large burden on public health [1]. Reduction of trauma-related mortality in high-income countries [2] has resulted in increased numbers of trauma survivors with long-term injury impact [3], including reduced health status (HS) [4]. An improved understanding of the quality of survival of patients is critically important for improving health care quality and in evaluating trauma care. Furthermore, it is important to understand short and long-term recovery patterns of HS in terms of injured patient characteristics and to identify predictors of outcome of seriously injured patients [5, 6].

Establishing recovery patterns in the short and long-term requires longitudinal data [7]. Non-fatal outcomes after trauma can be assessed with an overall measure of HS. HS includes patients' physical functioning, state of mind and social activities [8]. In general, trauma has a large impact on HS [4, 9-12], but large variations between patients have been observed [11, 12].

In addition, several relevant non-fatal outcomes after trauma may be assessed more specifically. These include psychological outcomes, such as anxiety and depression. Psychological problems are often reported among trauma patients [13-18] and are associated with worse HS [9, 16].

The fact that trauma has an impact on diverse aspects of patient health, illustrates that a multidimensional approach is necessary for a comprehensive understanding of non-fatal outcomes after trauma. This also allows studying the mutual relations between non-fatal outcomes. Using a multidimensional approach to measure outcomes including HS and symptoms of depression, anxiety and post-traumatic stress will result in a comprehensive understanding of non-fatal outcomes after trauma. In addition, to assess prognostic factors for a poor outcome~~,~~ it is important to cover the entire spectrum of the trauma population without exclusion of particular patient groups (e.g. elderly). The number of longitudinal cohort studies that examine multiple non-fatal outcomes in a large sample with a broad inclusion of type and severity of injury is limited [12, 19-23]. Most studies start measuring outcomes at least 3 months after trauma, resulting in little knowledge about the real short-term consequences [11, 24-27].

The overall aim of the Brabant Injury Outcome Surveillance (BIOS), a population based longitudinal study, is to provide more insight into recovery patterns and determinants of non-fatal outcomes after trauma. The aims of this population-based study are 1. to identify prognostic factors for decreased short, mid and long-term HS and ~~to describe the 2 year recovery patterns of HS and~~

~~psychological outcome for different categories of trauma patients and~~ 2. to describe the 2 year recovery patterns of HS and psychological outcome for different categories of trauma patients.~~to identify prognostic factors for decreased short, mid and long-term HS~~. This information is important for understanding the short- and long-term recovery patterns and for best informing provision of trauma care to injured patients with long-term disability.

Methods

Study design and participants

Data were obtained from the BIOS. The BIOS-study is a prospective observational cohort study in which HS and psychological outcomes are assessed in injured patients in the first 24 months after trauma. The methods of the BIOS have been described in detail in a published research protocol (doi: 10.1136/injuryprev-2016-042032) [28].

Recruitment occurred in all ten hospitals of the Noord-Brabant region (the Netherlands) from August 2015 until November 2016. Adults (≥18 years) who visited an emergency department ≤48 hours after trauma and who were admitted to the hospital, were invited to participate. All types of injuries were included~~,~~ regardless of the intent or severity of the injury. Patients who died between hospital discharge and the first week post-trauma, non-Dutch speaking patients, patients with no permanent address or patients with a pathological fracture were excluded. A proxy informant (caregiver or family member) was asked to complete the self-administered questionnaires if the patient was incapable of participating in the BIOS study him- or herself. Proxy informants were invited to enroll in the study 1 month post-trauma. Informal caregivers (e.g. family members) and paid caregivers (e.g. nurses) were allowed to function as proxy informants.

The Brabant Trauma Registry (BTR) compiles pre-hospital and hospital data of all trauma patients admitted after presentation to the ED in the Noord-Brabant region. ~~Before the data of the BTR and data of the BIOS-study were merged, the researchers randomly controlled the data of the trauma registry.~~ Quality of the data of the BTR and BIOS was checked on outliers and completeness by a trauma coordinator and researcher respectively. Furthermore, data from a sample of the trauma registry was checked manually by a trauma surgeon.

The study was approved by the Medical Ethics Committee Brabant, the Netherlands (project numbers NL50258.028.14 and NW2016-09). Prior to participation, participants signed an informed consent form.

Data collection

Questionnaires were sent at 1 week and 1, 3, 6, 12 and 24 months after trauma. Based on the participants' preference, follow-up questionnaires were either completed by paper and pencil or digitally. The questionnaires collected data on general patient characteristics (date of birth, gender), self-reported comorbidities (by using a modified version of the Cumulative Illness Rating Scale [29]), self-reported HS (i.e. EuroQol-5D-3L (EQ-5D-3L) [30], the Health Utilities Index (HUI) Mark 2 and Mark 3 [31]) and self-reported psychological functioning (i.e., Hospital Anxiety and Depression Scale (HADS) [32] and the Impact of Event Scale (IES) [33]). Proxy informants did not complete questionnaires regarding psychological outcome.

To increase the response rate, patients who did not complete a questionnaire up until 3 or 6 months post-trauma were asked to complete a short version of the BIOS-questionnaire. Patients who completed the shortened questionnaire included those who could not be reached by phone and did not return a BIOS questionnaire. In this short questionnaire, educational level, comorbidities, the EQ-5D-3L and the IES were included.This short questionnaire did not include proxy assessments. In the shortened questionnaire, pre-injury HS was not collected.

If participants did not complete the questionnaire, they were not excluded from the study but they were still invited at the subsequent time points.

Outcome measures

The EQ-5D and HUI are used in various studies measuring HS after trauma [9, 11, 24, 26, 34-39]. The EQ-5D provides valid results for trauma patients when it is completed by a proxy informant [40]. Moreover, a combination of the EQ-5D and the HUI is recommended for use in trauma patients since the combination of these measures covers all relevant dimensions of health [37, 41].

The EQ-5D consists of the EQ-5D descriptive system and the EQ-visual analogue scale (EQ-VAS). The EQ-5D comproisess? the following five dimensions: 'mobility', 'self-care', 'usual activities', 'pain/discomfort' and 'anxiety/depression'. Each dimension can be scored as 'no problems', 'moderate

problems' or 'severe problems' [30]. A scoring algorithm is available by which each HS description can be expressed as a summary score. This summary score ranges from 0 for death and 1 for full health and can be interpreted as a judgment on the relative desirability of an HS compared with perfect health. A summary score of these five dimensions (EQ-5D utility) can be calculated by using the Dutch tariffs [42]. The EQ-VAS is a vertical visual analogue scale with 0 indicating the worst imaginable health state and 100 indicating the best imaginable health state. The EQ-5D and EQ-VAS were also measured pre-injury, by asking participants 1 week or 1 month and proxy informants 1 month after the trauma for the patients' HS before sustaining the injury. The EQ-VAS was not included in the short questionnaire.

The HUI is a self-administered HS questionnaire that covers the main health domains that are affected by injury, with a particular focus on functional capacities. The HUI consists of 15 questions, classifying respondents into either the HUI2 or HUI3 health states [31]. Single-attribute and overall HS utility scores are calculated using the respective HUI2 and HUI3 utility functions. The results of the HUI questionnaires are converted by an algorithm into the levels of the complementary HUI2 and HUI3 classification system to form seven-element and eight-element health state vectors. From these vectors, single-attribute and overall health state utility scores are calculated [31].

For both the EQ-5D and the HUI, a scoring algorithm is used in which a score of 1 represents full health, 0 represents death and negative values indicate a HS of worse than death [30, 31].

The Hospital Anxiety and Depression Scale (HADS) was used to assess symptoms of anxiety and depression [32]. The HADS consists of 14 questions, 7 for symptoms of anxiety (HADSA) and 7 for depressive symptoms (HADSD). All questions have a 4-point response scale (0-3) and the scores for both subscales ranged from 0 to 21. A higher subscale score indicates greater severity of symptoms for anxiety and depression with a subscale value of ≥11 indicating a probable case (i.e., clinical symptoms) [32].

The IES was used to assess self-reported symptoms of post-traumatic stress [33]. The IES consists of 15 items of which the patient could use a 4-point scale (0=not at all, 1=rarely, 3=sometimes and 5=often) to determine whether the statement is present during the last seven days. The IES measures intrusive re-experience of the trauma and avoidance of trauma-related stimuli. A total sum score for the IES could be calculated ranging from 0 to 75. A score of ≥35 is considered as having symptoms of post-traumatic stress [33].

Prognostic factors

Hospital length of stay (LOS), admission to an Intensive Care Unit (ICU) and type and severity of injury were collected from the Brabant Trauma Registry and merged with the BIOS-data. The Abbreviated Injury Scale (AIS) codes (AIS-90, update 2008) [43] were used to create 14 injury group classifications (e.g. hip fracture, severe abdominal injury) representing the most common types of injuries (see S1 Table). Patients who suffer multiple injuries could be classified into one or more injury group classifications.

Trauma severity was based on the Injury Severity Score (ISS). The ISS is based on the square of the highest Abbreviated Injury Scales (AIS) scores of the three most severely injured body regions with a range of 1 to 75. An ISS of ≥16 is considered severely injured [43].

To determine socio-economic status (SES), educational level was used. Educational level was categorized into three levels; low (primary education, preparatory secondary vocational education or without diploma), middle (university preparatory education, senior general secondary education or senior secondary vocational education and training), and high (academic degree or university of applied science).

Statistical analyses

Patients were included in the analyses if they completed a questionnaire for at least one of the predetermined time points. For the non-responders of the BIOS, we could not obtain educational level. Therefore, in the non-responders analysis, status scores from 2014 were used as a proxy to indicate SES. Status scores were based on the mean income, % of people with a low income, % of people with low educational level and % of unemployed people in the neighborhood. In 2014, the mean status score in the Netherlands was 0.28 [44]. A lower status score indicates a lower SES whereas a higher status score indicates a higher SES. Responders and non-responders were compared on age, gender, status score, ISS, type of trauma, LOS and admission to an ICU using Mann-Whitney U tests and Chi-square tests (χ^2).

Means and standard deviations (SDs) of the EQ-5D-3L, HUI2, HUI3, HADSD, HADSA and IES summary scores were calculated and reported for the total BIOS population and for the different subcategories.

Multiple imputation was conducted with the Multivariate Imputation by Chained Equations procedure [45] to handle missing baseline characteristics and sum scores of the questionnaires due to missing item scores (see S2). The dataset was imputed 15 times with 5 iterations. Sensitivity analysis was performed in which only complete cases were included to compare results with the imputed datasets. S3 shows the differences between the original and imputed data.

Score options from each dimension of the EQ-5D were dichotomized into 0=‘no problems’ and 1=‘moderate problems’/‘severe problems’.

Four linear mixed models [46] with a random intercepts were performed to assess longitudinal association between prognostic factors and HS over the 24 months after trauma, which were divided into short-term (1 week and 1 month), mid-term (3 and 6 months) and long-term (12 and 24 months) associations. HS was measured with the EQ-5D-3L summary score.

The results were considered statistically significant at a level of $p<0.05$. All analyses were conducted in SPSS V.24 (Statistical Package for Social Sciences, Chicago, Illinois, USA), except of the multiple imputation which was performed in R version 3.4.0 (The R Project for Statistical Computing).

Results

BIOS cohort

During the inclusion period of the BIOS, a total of 10,227 patients were hospitalized because of trauma in one of the participating study centers. Patients were excluded if they did not speak the Dutch language (n=194), had no permanent address (n=32), died during their hospital stay within the first week after trauma (n=219) or had other reasons (n=8) (e.g., living abroad). Thus, 9,774 patients were eligible for participation in the BIOS, of whom 4,883 patients provided informed consent and were included (50% response rate). Of these 4,883 participants, 1,099 filled out the shortened questionnaires (see Fig 1).

At 1 week and 1, 3, 6, 12 and 24 months following the trauma, we collected data from of 1,776, 2,971, 3,109, 3,418, 3,105 and 2,734 participants (36.4%, 60.8%, 63.7%, 69.9%, 63.6% and 56.0%, respectively, of the study population) (see Fig 1). A total of 1,105 participants (22.6% of the study population) completed all BIOS questionnaires at each time point. In addition, data on pre-injury

HS were obtained from 3,366 participants (69% of the study population). After the first week assessment, missing questionnaires were the result of non-response (i.e., patients who had provided no data at any of the previous time points) and loss to follow-up (i.e., patients who had provided data for at least one of the previous time points). The main reason for participants to be lost to follow-up during the study period was that completing the questionnaires was too time consuming. Elderly, participants with low educational levels, longer hospital LOS, moderate injury (ISS 9-15), a hip fracture, severe traumatic brain injury (TBI) and those with severe abdominal trauma showed lower response rates to the 1 week questionnaire but provided data thereafter. In the BIOS, patients aged 18-24 and those who recovered completely were most likely to be lost to follow-up ~~FU~~.

-insert Fig1 here-

Study population

The median age of the study population was 68 years (IQR 53-80) (Table 1). Responders had a median ISS of 5 (IQR [4-9]) and a large part of the population reported comorbidities. Mild TBI (27.1%) and hip fracture (25.9%) were the most common types of trauma among the participants included in the BIOS. The majority of the participants (n=2,562, 52.5%) had a low educational level. A total of 407 participants (8% of the study population) were represented by a proxy informant.

Compared to the non-responders, participants were more severely injured and had a higher probability of being admitted to the ICU. In addition, responders had a higher median status score (based on the mean income, % of people with a low income, % of people with low educational level and % of unemployed people in the neighborhood) compared to the general Dutch population (mean 0.28) and compared to the median status score of the non-responders (median 0.33, min. score -3.03, max. score 2,58). Patients aged 18-44 and ≥85 years showed relatively low response rates (35%-40% and 39%, respectively). Patients with minor injuries (ISS 1-3) revealed a low response rate (46%), as well as patients with a hospital LOS of ≤2 or ≥15 days (46% and 45%, respectively).

Table 1. Characteristics of responders and non-responders of the Brabant Injury Outcome Surveillance.

Characteristics	Responders (n=4,883) %	Non-responders (n=4,891) %
**Gender (male)**	2,329 (47.7%)	2,407 (49.0%)
**Median age (yrs)**	68 (IQR 53-80)	70 (IQR 46-84)
18-24	217 (4.4%)	400 (8.2%)
25-44	516 (10.6%)	767 (15.7%)
45-64	1,364 (27.9%)	1,006 (20.6%)

65-74	963 (19.7%)	563 (11.5%)
75-84	1,102 (22.6%)	1,030 (21.1%)
≥85	721 (14.8%)	1,125 (23.0%)
Missing	0 (0.0%)	0 (0.0%)
**Median SES status score**	0.33 (IQR -0.24-0.84)	0.13 (IQR -0.36-0.73)
Missing	60 (1.2%)	68 (1.4%)
**Median days hospital LOS**	4 (IQR 2-8)	4 (IQR 2-8)
≤2	1,325 (27.1%)	1,528 (31.2%)
3-7	1,944 (39.8%)	1,642 (33.6%)
8-14	937 (19.2%)	911 (18.6%)
≥15	346 (7.1%)	421 (8.6%)
Missing	331 (6.8%)	389 (8.0%)
**Type of injury**		
Pelvic injury	293 (6.0%)	151 (3.1%)
Hip fracture	1,266 (25.9%)	1,099 (22.5%)
Tibia, complex foot or femur fracture	569 (11.7%)	505 (10.3%)
Shoulder and upper arm injury	473 (9.7%)	417 (8.5%)
Radius, ulna or hand fracture	308 (6.3%)	283 (5.8%)
Mild TBI	1,324 (27.1%)	1,443 (29.5%)
Serious TBI	126 (2.6%)	130 (2.7)
Severe TBI	77 (1.6%)	77 (1.6)
Facial fracture	249 (5.1%)	303 (6.2%)
Thoracic injury	198 (4.1%)	162 (3.3%)
Rib fracture	541 (11.1%)	398 (8.1%)
Mild abdominal injury	87 (1.8%)	89 (1.8%)
Severe abdominal injury	36 (0.7%)	30 (0.6%)
Spinal cord injury	27 (0.6%)	10 (0.2%)
Stable vertebral fracture or disc injury	301 (6.2%)	249 (5.1%)
**Injury severity**	5 (IQR 4-9)	5 (IQR 2-9)
ISS 1-3	1,145 (23.4%)	1,360 (27.8%)
ISS 4-8	1,597 (32.7%)	1,320 (27.0%)
ISS 9-15	1,857 (38.0%)	1,627 (33.3%)
ISS ≥16	239 (4.9%)	194 (4.0%)
Missing	45 (0.9%)	390 (8.0%)
**ICU-admission (yes)**	358 (7.3%)	292 (6.0%)
Missing	0 (0.0%)	0 (0.0%)

Abbreviations:SES, social-economic status; ICU, intensive care unit; ISS, Injury Severity Score; IQR, Interquartile range; LOS, length of stay; TBI, traumatic brain injury; yrs, years.

Health status

The mean EQ-5D-3L summary score increased from 0.49 (SD 0.32) at 1 week post-trauma to 0.77 (SD 0.26) at 24 months post-trauma. The mean pre-injury EQ-5D score was 0.85 (SD 0.23). In addition, the mean (SD) HUI2 and HUI3 scores increased from 0.61 (0.22) and 0.38 (0.31) at 1 week post-trauma to 0.77 (0.21) and 0.62 (0.35) at 24 months post-trauma, respectively (see Table 2). With regard to the individual domains of the EQ-5D, trauma patients reported various problems on the 'mobility, 'usual activities' and 'pain/discomfort' dimensions during the 24 months of follow-up (see Fig 2). In addition, during the 24 months, the prevalence of problems on all dimensions of the EQ-5D decreased, but remained higher at 24 months compared to pre-injury (46% and 32%, respectively for mobility, 23% and 16%, respectively for self-care, 44% and 26%, respectively for usual activities, 52% and 32%, respectively for pain/discomfort and 22% and 16%, respectively for anxiety/depression).

Table 2. Mean (SD) summary scores of self-reported health status and psychological outcomes up to 2 years post-trauma.

Time post-trauma	EQ-5D-3L*		HUI2**		HUI3**		HADSA***		HADSD***		IES****	
	N	Mean (SD)	N	Mean (SD)	N	Mean (SD)	N	Mean (SD)	N	Mean (SD)	N	Mean (SD)
1 week	1,776	0.49 (0.32)	1,776	0.61 (0.22)	1,776	0.38 (0.31)	1,776	4.92 (3.98)	1,776	5.00 (4.28)	1,776	14.80 (15.80)
1 month	2,971	0.56 (0.30)	2,971	0.67 (0.22)	2,971	0.45 (0.35)	2,644	4.81 (3.95)	2,644	4.77 (4.17)	2,644	14.44 (15.73)
3 months	3,131	0.69 (0.27)	2,735	0.72 (0.22)	2,735	0.53 (0.35)	2,423	4.57 (3.80)	2,423	4.24 (4.02)	2,819	12.75 (15.47)
6 months	3,433	0.74 (0.25)	2,442	0.75 (0.22)	2,442	0.58 (0.35)	2,191	4.21 (3.79)	2,191	3.91 (4.01)	3,182	11.42 (15.28)
12 months	3,151	0.76 (0.25)	2,391	0.76 (0.22)	2,391	0.60 (0.36)	2,165	4.32 (3.78)	2,165	3.74 (3.97)	2,925	10.98 (14.98)
24 months	2,734	0.77 (0.26)	2,137	0.77 (0.21)	2,137	0.62 (0.35)	1,990	4.31 (3.76)	1,990	3.62 (3.87)	2,587	10.35 (14.72)

*Completed by total study population. **Not administered to patients who completed only the short questionnaire. ***Not administered to proxy participants or to patients who completed only the short questionnaire. ****Not administered to patients aged ≥65 with a hip fracture who completed only the short questionnaire.

Range of EQ-5D-3L: 0-1. Mean EQ-5D-3L of the general Dutch population: 0.87 [47].

Range of HUI 2/3: 0-1

Range of HADSA and HADSD: 0-21

Range of IES: 0-75

Abbreviations: SD, standard deviation; EQ-5D, EuroQol-5D-3L; HUI2/3, Health Utilities Index Mark 2/3; HADS, Hospital Anxiety and Depression Scale; HADSA, Hospital Anxiety and Depression scale, subscale anxiety; HADSD, Hospital Anxiety and Depression scale, subscale depression; IES, Impact of Event Scale.

-insert Fig2 here-

Psychological outcomes

The mean summary scores (SD) of the HADSA (clinical symptoms of anxiety), HADSD (clinical symptoms of depression) and IES (symptoms of post-traumatic stress) slightly decreased from 4.92 (3.98), 5.00 (4.28) and 14.80 (15.80), respectively, at 1 week post-trauma to 4.31 (3.76), 3.62 (3.87) and 10.35 (14.72), respectively, at 24 months post-trauma (see Table 2).

The prevalence rates of clinical symptoms of anxiety (HADSA≥11), depression (HADSD≥11) and post-traumatic stress (IES≥35) reported at 1 week post-trauma were 10.2%, 12.3% and 13.5%, respectively, and showed a small decrease over time to 7.8%, 6.8% and 11.0% at 24 months post-trauma, respectively.

The results revealed that patients with symptoms of post-traumatic stress (IES≥35) showed worse outcomes on the EQ-5D-3L than patients with no symptoms of post-traumatic stress (Fig. 3).

-insert Fig3 here-

Prognostic factors of health status

Overall, HS measured as with the EQ-5D-3L increased over at least up until 6 months for all groups of patients and stabilized between 6 and 12 months post-trauma for most groups (see Table 3). Female patients had a lower HS compared to males at every time point.

At all time points, patients aged 85 and older had the lowest HS compared to the other age categories. At 3 and 6 months, all patient groups between 25 and 74 years reported the same HS whereas patients aged between 18 and 24 reported a higher EQ-5D summary score. HS stabilized at 6 or 12 months for every age group, except for patients between 25 and 44 years for whom HS increased further.

Except for 1 week, ~~at all time points~~ patients with a high educational level had the highest HS. With an increasing number of comorbidities, HS decreased. Patients with moderate injuries (ISS 9-15) showed on~~,~~ almost each time point the lowest HS. At 1 week, severely injured patients (ISS ≥16) showed the lowest mean HS (0.28, SD 0.35).

Patients with the longest hospital LOS~~stay~~ (≥15 days) had the lowest mean HS at all time points, ranging from 0.28 (SD 0.34) at 1 week up to 0.62 (SD 0.28) at 24 months after trauma.

Table 3. Mean (SD) of the self-reported health status for patient and injury characteristics as measured with the EQ-5D-3L.

		Mean (SD) EQ-5D-3L summary score						
**Characteristics**		**Pre-injury**	**1 week**	**1 month**	**3 months**	**6 months**	**12 months**	**24 months**
**Gender**	**Male**	0.90 (0.19)	0.54 (0.32)	0.62 (0.28)	0.74 (0.25)	0.79 (0.23)	0.83 (0.21)	0.84 (0.21)
	**Female**	0.80 (0.25)	0.43 (0.31)	0.50 (0.30)	0.64 (0.28)	0.69 (0.26)	0.70 (0.27)	0.72 (0.28)
**Age (yrs)**	**18-24**	0.95 (0,13)	0.50 (0.32)	0.63 (0.24)	0.79 (0.24)	0.85 (0.21)	0.86 (0.21)	0.86 (0.22)
	**25-44**	0.95 (0.12)	0.45 (0.30)	0.59 (0.29)	0.74 (0.24)	0.79 (0.24)	0.84 (0.22)	0.87 (0.19)
	**45-64**	0.91 (0.17)	0.49 (0.31)	0.60 (0.27)	0.73 (0.24)	0.79 (0.21)	0.83 (0.21)	0.83 (0.22)
	**65-74**	0.88 (0.20)	0.51 (0.31)	0.62 (0.28)	0.73 (0.24)	0.79 (0.22)	0.80 (0.22)	0.81 (0.23)
	**75-84**	0.78 (0.26)	0.52 (0.33)	0.53 (0.31)	0.66 (0.27)	0.70 (0.25)	0.70 (0.26)	0.70 (0.28)
	**≥85**	0.63 (0.28)	0.40 (0.33)	0.39 (0.32)	0.50 (0.29)	0.55 (0.29)	0.57 (0.29)	0.56 (0.30)
**Educational level**	**Low**	0.78 (0.27)	0.49 (0.33)	0.52 (0.31)	0.65 (0.28)	0.70 (0.27)	0.71 (0.27)	0.72 (0.28)
	**Middle**	0.90 (0.17)	0.50 (0.31)	0.59 (0.29)	0.71 (0.26)	0.77 (0.24)	0.80 (0.23)	0.81 (0.23)
	**High**	0.93 (0.13)	0.48 (0.30)	0.62 (0.26)	0.75 (0.23)	0.80 (0.21)	0.84 (0.20)	0.86 (0.20)
**Comorbidity**	**0**	0.96 (0.12)	0.53 (0.30)	0.65 (0.26)	0.77 (0.23)	0.83 (0.19)	0.86 (0.18)	0.87 (0.18)
	**1**	0.85 (0.21)	0.49 (0.32)	0.56 (0.29)	0.69 (0.25)	0.75 (0.24)	0.77 (0.24)	0.78 (0.25)
	**2**	0.75 (0.24)	0.45 (0.30)	0.47 (0.31)	0.61 (0.29)	0.66 (0.26)	0.67 (0.27)	0.68 (0.28)
	**≥3**	0.64 (0.29)	0.40 (0.33)	0.42 (0.32)	0.56 (0.29)	0.58 (0.29)	0.59 (0.29)	0.59 (0.29)
**ISS**	**1-3**	0.86 (0.22)	0.63 (0.29)	0.69 (0.27)	0.78 (0.23)	0.79 (0.24)	0.79 (0.25)	0.81 (0.25)
	**4-8**	0.89 (0.20)	0.46 (0.31)	0.56 (0.29)	0.71 (0.25)	0.77 (0.23)	0.80 (0.22)	0.81 (0.23)
	**9-15**	0.80 (0.26)	0.43 (0.30)	0.50 (0.30)	0.63 (0.28)	0.68 (0.27)	0.70 (0.28)	0.72 (0.28)
	**≥16**	0.90 (0.18)	0.37 (0.35)	0.50 (0.31)	0.65 (0.30)	0.74 (0.25)	0.77 (0.24)	0.77 (0.25)
**LOS (days)**	**≤2**	0.91 (0.18)	0.61 (0.29)	0.70 (0.25)	0.81 (0.21)	0.83 (0.20)	0.84 (0.22)	0.86 (0.21)
	**3-7**	0.85 (0.23)	0.47 (0.30)	0.55 (0.28)	0.69 (0.25)	0.74 (0.24)	0.77 (0.24)	0.79 (0.24)
	**8-14**	0.78 (0.27)	0.31 (0.31)	0.46 (0.29)	0.59 (0.29)	0.65 (0.27)	0.68 (0.28)	0.67 (0.29)
	**≥15**	0.74 (0.28)	0.28 (0.34)	0.32 (0.31)	0.50 (0.29)	0.58 (0.28)	0.60 (0.28)	0.62 (0.28)

Range of EQ-5D-3L: 0-1. Mean EQ-5D-3L of the general Dutch population: 0.87.
Abbreviations: SES, socio-economic status; SD, standard deviation; EQ-5D, EuroQol-5D-3L; ISS, Injury Severity Score; LOS, length of hospital stay; yrs, years

After adjustment for confounding factors,~~-~~ short-term (1 week and 1 month) prognostic factors for a significant lower EQ-5D summary score were female gender, a higher number of comorbidities, a longer LOS, a higher ISS, pelvic injury, tibia/complex foot or femur fracture, radius/ulna/hand fracture, shoulder/upper arm injury, rib fracture, spinal cord injury and stable vertebral fracture/disc injury (see Table 4). Mid-term (3 and 6 months)

prognostic factors were a higher number of comorbidities, an ISS between 4 and 15, a longer LOS, radius/ulna/hand fracture, tibia/complex foot or femur fracture, severe TBI, spinal cord injury and stable vertebral fracture/disc injury. Long-term (12 and 24 months) prognostic factors for a lower HS were: an age 75 and above, 2 or more comorbidities, a longer LOS, tibia/complex foot or femur fracture, spinal cord injury and stable vertebral fracture/disc injury were prognostic factors for a lower HS. A high educational level was associated with higher HS in the long-term analysis.

Table 4. Multivariable longitudinal analysis of short, mid and long-term prognostic factors for decreased health status as measured with the EQ-5D-3L.

			**Total (1 week-24 months)**	**Short-term (1 week-1 month)**	**Mid-term (3-6 months)**	**Long-term (12-24 months)**
		**Number of patients**	*** Beta (95% C.I.)**	*** Beta (95% C.I.)**	*** Beta (95% C.I.)**	*** Beta (95% C.I.)**
**Characteristics**		**4,809**	**n=4,809**	**n=3,314**	**n=4,048**	**n=3,422**
**Gender**	Male	2,291	ref	ref	ref	ref
	Female	2,518	-0.05 (-0.06; -0.04)	-0.06 (-0.08; -0.05)	-0.05 (-0.06;-0.03)	-0.06 (-0.08; -0.05)
**Age (yrs)**	18-44	721	ref	ref	ref	ref
	45-64	1,345	0.04 (0.02; 0.05)	0.06 (0.04; 0.09)	0.02 (-0.00; 0.04)	0.01 (-0.01; 0.03)
	65-74	956	0.09 (0.08; 0.11)	0.14 (0.11; 0.17)	0.07 (0.05; 0.09)	0.04 (0.02; 0.06)
	≥75	1,787	0.02 (0.01; 0.04)	0.09 (0.06; 0.12)	-0.01 (-0.03; 0.01)	-0.05 (-0.07; -0.02)
**Educational level**	Low	2,637	ref	ref	ref	ref
	Middle	1,280	0.02 (0.00; 0.03)	0.01 (-0.01; 0.03)	0.01 (-0.00; 0.02)	0.03 (0.01; 0.04)
	High	893	0.03 (0.02; 0.05)	0.02 (-0,01; 0,04)	0.04 (0.02; 0.05)	0.05 (0.04; 0.07)
**Number of comor-bidities**	None	1,801	ref	ref	ref	ref
	1	1,426	-0.06 (-0.07; -0.05)	-0.06 (-0.08; -0.04)	-0.06 (-0.07; -0.04)	-0.05 (-0.07; -0.04)
	≥2	1,582	-0.15 (-0.16; -0.14)	-0.14 (-0.17; -0.12)	-0.14 (-0.16;-0.13)	-0.16 (-0.18; -0.15)
**ISS**	1-3	1,139	ref	ref	ref	ref
	4-8	1,596	0.00 (-0.01; 0.02)	-0.03 (-0.06; -0.01)	0.01 (-0.05; 0.03)	0.03 (0.01; 0.05)
	9-15	1,838	-0.02 (-0.04; -0.00)	-0.06 (-0.09; -0.02)	-0.02 (-0.04; -0.01)	0.00 (-0.02; 0.03)
	≥16	235	0.01 (-0.01; 0.04)	-0.02 (-0.08; 0.04)	0.01 (-0.04; 0.05)	0.04 (-0.01; 0.08)
**LOS**	≤2	1,425	ref	ref	ref	ref
	3-7	2,053	-0.05 (-0.06; -0.04)	-0.08 (-0.10; -0.06)	-0.06 (-0.07; -0.04)	-0.03 (-0.04; -0.01)
	8-14	975	-0.11 (-0.12; -0.09)	-0.15 (-0.18; -0.12)	-0.11 (-0.13; -0.09)	-0.08 (-0.11; -0.06)
	≥15	357	-0.20 (-0.22; -0.18)	-0.24 (-0.28; -0.20)	-0.20 (-0.23; -0.17)	-0.17 (-0.20; -0.14)
**Injury**	Pelvic injury	286	-0.05 (-0.07; -0.03)	-0.13 (-0.16; -0.10)	-0,01 (-0.04; 0.01)	0.00 (-0.02; 0.03)
	Hip fracture	1,242	-0.02 (-0.04; -0.01)	-0.02 (-0.05; 0.01)	-0.03 (-0.05; -0.00)	-0.02 (-0.05; 0.00)
	Tibia, complex foot or femur fracture	561	-0.05 (-0.06; -0.04)	-0.11 (-0.14; -0.08)	-0.05 (-0.07; -0.02)	-0.02 (-0.04; 0.00)
	Shoulder and upper arm injury	469	-0.03 (-0.04; -0.01)	-0.07 (-0.10; -0.05)	-0.02 (-0.04; 0.00)	-0.00 (-0.02; 0.02)
	Radius, ulna or hand fracture	304	0.00 (-0.02; 0.02)	-0.03 (-0.06; 0.00)	0.00 (-0.02; 0.03)	0.02 (-0.00; 0.05)
	Mild TBI (AIS 1-2)	1,302	0.03 (0.02; 0.04)	0.06 (0.04; 0.08)	0.03 (0.02; 0.05)	0.02 (-0.00; 0.03)
	Serious TBI (AIS 3)	125	0.04 (0.01; 0.07)	0.07 (0.02; 0.13)	0.05 (0.01; 0.10)	0.01 (-0.03; 0.05)

	Severe TBI (≥ 4)	76	-0.03 (-0.06; 0.02)	-0.02 (-0.10; 0.07)	-0.06 (-0.12; 0.00)	-0.01 (-0.07; 0.05)
	Facial fracture	243	0.02 (0.01; 0.04)	0.05 (0.01; 0.09)	0.02 (-0.01; 0.04)	0.01 (-0.01; 0.04)
	Thoracic injury	198	0.04 (0.02; 0.06)	0.05 (0.01; 0.10)	0.04 (0.02; 0.07)	0.03 (-0.01; 0.06)
	Rib fracture	533	0.01 (-0.00; 0.03)	-0.01 (-0.04; 0.01)	0.03 (0.01; 0.05)	0.01 (-0.01; 0.04)
	Mild abdominal injury	85	0.02 (-0.02; 0.05)	0.04 (-0.02; 0.10)	0.01 (-0.04; 0.05)	0.02 (-0.03; 0.07)
	Severe abdominal injury	36	0.03 (-0.02; 0.08)	-0.05 (-0.15; 0.05)	0.06 (-0.04; 0.13)	0.05 (-0.02; 0.13)
	Spinal cord injury	27	-0.11 (-0.17; -0.05)	-0.10 (-0.22; 0.03)	-0.12 (-0.20; -0.03)	-0.15 (-0.23; -0.07)
	Stable vertebral fracture or disc injury	296	-0.05 (-0.07; -0.03)	-0.08 (-0.12; -0.05)	-0.05 (-0.08; -0.03)	-0.03 (-0.06; -0.01)

** Mixed models, adjusted for all other variables*
Beta: mean increase in EQ-5D-3L score (improvement of Health Status) compared to the reference category.
Range of EQ-5D-3L: 0-1. Mean EQ-5D-3L of the general Dutch population: 0.87 [47]. ~~easures how strong each predictor variable influences the dependent variable.~~
Abbreviations: ISS, Injury Severity Score; AIS, Abbreviated Injury Scale; LOS, Hospital lenght of stay in days; TBI, traumatic brain injury; yrs=years

Formatted: Adjust space between Latin and Asian text, Adjust space between Asian text and numbers

Discussion

This study describes HS and psychological outcomes over 24 months after trauma. HS markedly improved during the 24 months after trauma, most of which occurred within the first 3 months. Compared to pre-injury HS, a large decrease in HS was found 1 week post-trauma. The prevalence rates of symptoms of anxiety and depression were relatively low. In contrast, symptoms of post-traumatic stress were highly prevalent and were present five times as often as compared with the Dutch general population [48]. Additionally, in line with the literature, having symptoms of post-traumatic stress were~~as~~ associated with a lower HS [27]. The addition of an assessment at 1 week post-trauma in the present study adds detailed insight into (baseline) functioning shortly after trauma. Therefore, it provides a more valid assessment of the magnitude of recovery thereafter.

Between 1 week and 3 months, the percentage of patients who reported problems on the ‘pain/discomfort’ and ‘self-care’ dimensions of the EQ-5D decreased steeply. For the ‘mobility’ and ‘usual activities’ dimensions, this decrease started 1 month after trauma. The percentage of patients who reported problems on the ‘anxiety and depression’ dimension was the highest at 1 month after trauma. Within 6 months post-trauma, patients showed the most recovery. From 6 months post-trauma onwards, little improvement in overall HS was found. The percentage of patients who reported improvements on the different EQ-5D domains increased through the 24 months of follow-up. However, the vast majority of trauma patients did not recover to their pre-injury HS. The mean EQ-5D-

3L summary score for the total Dutch adult population is considered 0.87 (SD 0.18) (for males 0.89 (SD 0.16) and for females 0.85 (SD 0.19)) [47].

At short-term (up to 1 month post-trauma) female gender, lower extremity, spine, shoulder and upper arm injuries, injury severity, comorbidities and a longer hospital stay were associated with lower HS. At mid-term (3 and 6 months), almost the same prognostic factors were significant and relevant, however, only injury severity seemed to be less important. In the long-term a greater age, two or more comorbidities, a longer hospital stay and only a few injuries (i.e., lower extremity fracture and spine injury) showed a significantly lower HS. The effect of injury severity seemed to fade over time. Spinal cord injury patients had the highest risk (long-term Beta -0,18, CI -0,27;-0,08) of a lower HS during both the short (not significant), mid and long-term post-trauma~~ periods~~. Middle and high educational levels were associated with a higher HS in the long term compared to those with low educational levels.

Most recovery in HS occurs up to 3 months post-trauma, which is in agreement with previous studies on this topic [9, 10, 19]. In this regard, the addition of an assessment at 1 week post-trauma in the present study adds detailed insight into (baseline) functioning shortly after trauma. ~~Thereby, it provides a more valid assessment of the magnitude of recovery. ~~Prior work also confirms the finding that a large proportion of patients have a considerably lower HS 1 year post-trauma compared to pre-injury HS [9, 11, 16] or compared to the HS of the general population [49].

In addition to the physical injury itself, other characteristics largely affect HS after trauma. This was~~,~~ ~~in ~~particularly i~~i~~n the long-~~ -~~term. This finding extends those of previous studies [15, 35], confirming that patients who were the most severely injured, were not necessarily those with the lowest HS.

In this study, the prevalence of symptoms of anxiety and depression was slightly higher compared to the prevalence of an anxiety disorder or depression in the general Dutch population (both disorders are estimated to be present in 10.0% of the Dutch population) [50, 51]. As a result, the prevalence of symptoms of anxiety and depression slightly decreased over time. Recent and comparable studies have documented a slightly higher prevalences of symptoms of anxiety and depression [9, 16].

The prevalence of symptoms of post-traumatic stress was high as compared with the Dutch population (11.0% at 2 years post-trauma versus 2.6-3.3%) [48]. Our prevalence rate of long-term

symptoms of post-traumatic stress is in line with previous research that also uses the ≥35 cut-off point for the IES [13]. However, the systematic review by Haagsma *et al.* [52] revealed prevalence of post-traumatic stress in hospitalized trauma patients that ranged from 30% (90% CI 27%-33%) within 3 months post-trauma to 6% (90% CI 4%-10%) at 1 year. Compared to those results, we found a lower prevalence of symptoms of post-traumatic stress early post-trauma whereas a higher prevalence was found at the 1 year follow-up. This discrepancy may be due to studies using various instruments and different cut-off points to indicate post-traumatic stress.

This study was conducted according to the recommended guidelines for measuring non-fatal outcomes after trauma [41]. The BIOS included a broad study population, measured both short-term and long-term outcomes, measured functioning prior to the trauma, included a large number of patient and injury-related characteristics and requested information from proxy informants for patients who were incapable of completing the set of questionnaires themselves. Recruitment for the BIOS occurred in all hospitals of the Dutch Noord-Brabant region, covering both urban and rural populations.

The inclusion of elderly~~–~~ individuals with a hip fracture in the current study improves the generalizability of the study findings. In this study, one out of four participants was aged 65 or older and had a hip fracture. We are aware that recovery patterns (due to comorbidities and functional decline) in the elderly population are different from recovery patterns in younger patients.

T~~However, t~~his study also has several limitations. First, there was selection bias since younger patients, elderly patients, patients with very minor injuries (ISS 1-3) and those with a low status score (used as a proxy to indicate SES~~,~~; a low status score indicate a lower SES-level) were less likely to participate. It is challenging to include these specific groups. Previous studies also reported lower response rates from younger and elderly patients [7, 10, 24, 35], from patients with minor injuries [10] and from those with low educational level [53, 54]. Second, only a selected group of patients (18% of the eligible population) completed the 1 week assessment. Since most recovery occurs within the first 3 months post-trauma, it is vital to examine very early recovery patterns. This study provides unique data since it incorporates the use of a standardized 1 week assessment in a comprehensive group of trauma patients. Nevertheless~~,~~ in most cases, non-responders at this time point felt too disabled to complete a long questionnaire. Therefore, this most likely led to an underestimations of the reported HS and psychological outcomes 1 week post-trauma. Third, there were many missing data, especially early post-trauma. However, we were able to impute missing sum scores in several cases. Fourth, we

did not correct for pre-injury HS in the longitudinal analyses since we were not able to collect pre-injury HS in all participants. -Fifth, we did not include a pre-injury assessment of the symptoms of anxiety or depression. Sixth, we cannot exclude change findings due to multiple testing. Change findings might have occurred for the results of the longitudinal analyses as presented in Table 4.

Given the acute nature of trauma, it is difficult to include patients soon after they experience trauma in order to examine very early recovery patterns. To increase the response rate and to reduce loss to follow-up, future research should minimize the large number of questionnaires that patients have to complete at each time point, especially early post-trauma. A promising technique includes computerized adaptive testing (CAT). In this already proven valid technique, tailored-made short and precise domain-specific data can be collected [55-58].

We are aware that recall bias and response shift most likely led to an overestimation of the pre-injury HS as measured in this study. However, to produce valid estimates of the health impact and the decrease in functioning after trauma, information on patients functioning prior to the trauma is crucial [59-62]. Future research should focus on the effects of recall bias and response shift on retrospectively collected data.

Implications for health-care

According to the literature, early recognition, treatment and monitoring of psychological problems improve non-fatal outcomes after trauma [16, 63-66]. Therefore, early screening and interventions to reduce symptoms of post-traumatic stress should be part of standard care. Furthermore in the long long-term, patients aged ≥75 years, patients with a longer length of hospital stay and patients with ≥2 comorbidities are more likely to have a poor HS. For these patients, standard aftercare should be extended to screen for remaining problems that the patients should address after their trauma. For example, e.g., by a follow-up appointment with a case manager.

Symptoms of post-traumatic stress were frequently reported in this study. Patients need to be better informed about the psychological problems they may experience after trauma. Health- care providers should not solely focus on the physical consequences but should also be aware of all other possible consequences that may occur after trauma. To achieve this, a more holistic approach towards the treatment of trauma patients should be aimed in which the patients' own perspectives on their recovery should play a crucial role.

Conclusion

Hospitalized trauma patients experience substantial reductions in HS and frequently report symptoms of post-traumatic stress. The results of this study should be interpreted with caution since younger patients, patients with very minor injuries and those with a lower status score (used as an proxy to indicate SES, a lower status score indicates a lower SES-level) were less likely to participate. Most improvements in HS and psychological symptoms occurred within the first 3 months post-trauma. After two years post trauma, the vast majority of trauma patients did not achieve their pre-injury HS. Recovery trajectories varied widely in which female gender, age ≥75 years, spinal cord injury, having more comorbidities, low educational level, a longer hospital stay and symptoms of post-traumatic stress were associated with a higher risk of decreased HS in the long-term after trauma. In the short-term, also several lower extremity injuries are prognostic factors for decreased HS.

Acknowledgements

Memberschip list of the BIOS-group: P.V. van Eerten (Maxima Medical Center, department of surgery, Eindhoven, the Netherlands), F.C. van Eijck (Bravis Hospital, department of surgery, Bergen op Zoom and Roosendaal, the Netherlands), H.J. van Geffen (Jeroen Bosch Hospital, department of surgery, ‘s-Hertogenbosch, the Netherlands), W.A. Haagh (St. Anna Hospital, department of surgery, Geldrop, the Netherlands), L.M. Poelhekke (Maasziekenhuis Pantein Hospital, department of surgery, Boxmeer, the Netherlands), J.B. Sintenie (Elkerliek Hospital, department of surgery, Helmond, the Netherlands, C.T. Stevens (Bernhoven Hospital, department of surgery, Uden, the Netherlands), A.H. van der Veen (Catharina Hospital, department of surgery, Eindhoven, the Netherlands), C.H. van der Vlies (Maasstad Hospital, department of surgery, Rotterdam, Eindhoven, D.I. Vos (Amphia Hospital, department of surgery, Breda, the Netherlands). Contact person of the BIOS-group: A.H. van der Veen: alexander.vd.veen@catharinaziekenhuis.nl

References

1. Murray CJ, Vos T, Lozano R, Naghavi M, Flaxman AD, Michaud C, et al. Disability-adjusted life years (DALYs) for 291 diseases and injuries in 21 regions, 1990-2010: a systematic analysis for the Global Burden of Disease Study 2010. Lancet. 2012;380(9859):2197-223. doi: 10.1016/S0140-6736(12)61689-4. PubMed PMID: 23245608.
2. WorldHealthOrganization. Injuries and violence, the facts. 2014.
3. Haider AH, Herrera-Escobar JP, Al Rafai SS, Harlow AF, Apoj M, Nehra D, et al. Factors Associated With Long-Term Outcomes After Injury: Results of the Functional Outcomes and Recovery After Trauma Emergencies (FORTE) Multicenter Cohort Study. Ann Surg. 2018. Epub 2018/12/15. doi: 10.1097/SLA.0000000000003101. PubMed PMID: 30550382.
4. Lyons RA, Kendrick D, Towner EM, Christie N, Macey S, Coupland C, et al. Measuring the population burden of injuries--implications for global and national estimates: a multi-centre prospective UK longitudinal study. PLoS Med. 2011;8(12):e1001140. doi: 10.1371/journal.pmed.1001140. PubMed PMID: 22162954; PubMed Central PMCID: PMCPMC3232198.
5. Bhalla K, Harrison J, Abraham J, Borse NN, Lyons R, Boufous S, et al. Data sources for improving estimates of the global burden of injuries: call for contributors. PLoS Med. 2009;6(1):e1. doi: 10.1371/journal.pmed.1000001. PubMed PMID: 19166263; PubMed Central PMCID: PMCPMC2628399.
6. Polinder S, Haagsma JA, Lyons RA, Gabbe BJ, Ameratunga S, Cryer C, et al. Measuring the population burden of fatal and nonfatal injury. Epidemiol Rev. 2012;34:17-31. doi: 10.1093/epirev/mxr022. PubMed PMID: 22113244.
7. Gabbe BJ, Simpson PM, Cameron PA, Ponsford J, Lyons RA, Collie A, et al. Long-term health status and trajectories of seriously injured patients: A population-based longitudinal study. PLoS Med. 2017;14(7):e1002322. doi: 10.1371/journal.pmed.1002322. PubMed PMID: 28678814; PubMed Central PMCID: PMCPMC5497942.
8. de Vries J. Quality of life assessment, in Assessment in behavorial medicine, A.J.J.M. Vingerhoets, Editor 2001, Brunner-Routledge: Hove. 2001.

Formatted: Dutch (Netherlands)

9. Kendrick D, Kelllezi B, Coupland C, Maula A, Beckett K, Morriss R, et al. Psychological morbidity and health-related quality of life after injury: multicentre cohort study. Qual Life Res. 2017;26(5):1233-50. doi: 10.1007/s11136-016-1439-7. PubMed PMID: 27785608; PubMed Central PMCID: PMCPMC5376395.

10. Meerding WJ, Looman CW, Essink-Bot ML, Toet H, Mulder S, van Beeck EF. Distribution and determinants of health and work status in a comprehensive population of injury patients. J Trauma. 2004;56(1):150-61. doi: 10.1097/01.TA.0000062969.65847.8B. PubMed PMID: 14749582.

11. Harcombe H, Langley J, Davie G, Derrett S. Functional status following injury: what recovery pathways do people follow? Injury. 2015;46(7):1275-80. doi: 10.1016/j.injury.2015.04.004. PubMed PMID: 25920372.

12. Polinder S, van Beeck EF, Essink-Bot ML, Toet H, Looman CW, Mulder S, et al. Functional outcome at 2.5, 5, 9, and 24 months after injury in the Netherlands. J Trauma. 2007;62(1):133-41. doi: 10.1097/TA.0b013e31802b71c9. PubMed PMID: 17215744.

13. van Delft-Schreurs CC, van Bergen JJ, van de Sande P, Verhofstad MH, de Vries J, de Jongh MA. A cross-sectional study of psychological complaints and quality of life in severely injured patients. Qual Life Res. 2014;23(4):1353-62. doi: 10.1007/s11136-013-0546-y. PubMed PMID: 24202637.

14. Haagsma JA, Polinder S, Olff M, Toet H, Bonsel GJ, van Beeck EF. Posttraumatic stress symptoms and health-related quality of life: a two year follow up study of injury treated at the emergency department. BMC Psychiatry. 2012;12:1. doi: 10.1186/1471-244X-12-1. PubMed PMID: 22230388; PubMed Central PMCID: PMCPMC3276433.

15. van Delft-Schreurs CC, van Bergen JJ, de Jongh MA, van de Sande P, Verhofstad MH, de Vries J. Quality of life in severely injured patients depends on psychosocial factors rather than on severity or type of injury. Injury. 2014;45(1):320-6. doi: 10.1016/j.injury.2013.02.025. PubMed PMID: 23510753.

16. Kellezi B, Coupland C, Morriss R, Beckett K, Joseph S, Barnes J, et al. The impact of psychological factors on recovery from injury: a multicentre cohort study. Soc Psychiatry Psychiatr Epidemiol. 2017;52(7):855-66. doi: 10.1007/s00127-016-1299-z. PubMed PMID: 27803978; PubMed Central PMCID: PMCPMC5504249.

17. Skogstad L, Toien K, Hem E, Ranhoff AH, Sandvik L, Ekeberg O. Psychological distress after physical injury: a one-year follow-up study of conscious hospitalised patients. Injury. 2014;45(1):289-98. doi: 10.1016/j.injury.2012.10.001. PubMed PMID: 23103082.

18. Wiseman TA, Curtis K, Lam M, Foster K. Incidence of depression, anxiety and stress following traumatic injury: a longitudinal study. Scand J Trauma Resusc Emerg Med. 2015;23:29. doi: 10.1186/s13049-015-0109-z. PubMed PMID: 25882818; PubMed Central PMCID: PMCPMC4389309.

19. Kendrick D, Vinogradova Y, Coupland C, Mulvaney CA, Christie N, Lyons RA, et al. Recovery from injury: the UK burden of injury multicentre longitudinal study. Inj Prev. 2013;19(6):370-81. doi: 10.1136/injuryprev-2012-040658. PubMed PMID: 23599210.

20. Langley J, Derrett S, Davie G, Ameratunga S, Wyeth E. A cohort study of short-term functional outcomes following injury: the role of pre-injury socio-demographic and health characteristics, injury and injury-related healthcare. Health Qual Life Outcomes. 2011;9:68. doi: 10.1186/1477-7525-9-68. PubMed PMID: 21849082; PubMed Central PMCID: PMCPMC3170567.

21. Derrett S, Davie G, Ameratunga S, Wyeth E, Colhoun S, Wilson S, et al. Prospective Outcomes of Injury Study: recruitment, and participant characteristics, health and disability status. Inj Prev. 2011;17(6):415-8. doi: 10.1136/injuryprev-2011-040044. PubMed PMID: 21724742.

22. Mackenzie EJ, Rivara FP, Jurkovich GJ, Nathens AB, Frey KP, Egleston BL, et al. The National Study on Costs and Outcomes of Trauma. J Trauma. 2007;63(6 Suppl):S54-67; discussion S81-6. doi: 10.1097/TA.0b013e31815acb09. PubMed PMID: 18091213.

23. Lyons RA, Towner EE, Kendrick D, Christie N, Brophy S, Phillips CJ, et al. The UK burden of injury study - a protocol. [National Research Register number: M0044160889]. BMC Public Health. 2007;7:317. doi: 10.1186/1471-2458-7-317. PubMed PMID: 17996057; PubMed Central PMCID: PMCPMC2225415.

24. Langley J, Davie G, Wilson S, Lilley R, Ameratunga S, Wyeth E, et al. Difficulties in functioning 1 year after injury: the role of preinjury sociodemographic and health characteristics, health care and injury-related factors. Arch Phys Med Rehabil. 2013;94(7):1277-86. doi: 10.1016/j.apmr.2013.02.012. PubMed PMID: 23439409.

25. Gabbe BJ, Simpson PM, Harrison JE, Lyons RA, Ameratunga S, Ponsford J, et al. Return to Work and Functional Outcomes After Major Trauma: Who Recovers, When, and How Well? Ann Surg. 2016;263(4):623-32. doi: 10.1097/SLA.0000000000001564. PubMed PMID: 26779977.

26. Gabbe BJ, Simpson PM, Lyons RA, Ameratunga S, Harrison JE, Derrett S, et al. Association between the number of injuries sustained and 12-month disability outcomes: evidence from the injury-VIBES study. PLoS One. 2014;9(12):e113467. doi: 10.1371/journal.pone.0113467. PubMed PMID: 25501651; PubMed Central PMCID: PMCPMC4263479.

27. Giummarra MJ, Casey SL, Devlin A, Ioannou LJ, Gibson SJ, Georgiou-Karistianis N, et al. Co-occurrence of posttraumatic stress symptoms, pain, and disability 12 months after traumatic injury. Pain Rep. 2017;2(5):e622. doi: 10.1097/PR9.0000000000000622. PubMed PMID: 29392235; PubMed Central PMCID: PMCPMC5777683.

28. de Jongh MA, Kruithof N, Gosens T, van de Ree CL, de Munter L, Brouwers L, et al. Prevalence, recovery patterns and predictors of quality of life and costs after non-fatal injury: the Brabant Injury Outcome Surveillance (BIOS) study. Inj Prev. 2016. doi: 10.1136/injuryprev-2016-042032. PubMed PMID: 27154507.

29. Linn BS, Linn MW, Gurel L. Cumulative illness rating scale. J Am Geriatr Soc. 1968;16(5):622-6. PubMed PMID: 5646906.

30. EuroQol G. EuroQol--a new facility for the measurement of health-related quality of life. Health Policy. 1990;16(3):199-208. PubMed PMID: 10109801.

31. Furlong W, Feeny D, Torrance G. Health Utilities Index (HUI) procedures manual: algorithm for determining HUI Mark 2 (HUI2)/Mark 3 (HUI3) health status classification levels, health states single-attribute level utility scores and overall health-related quality of life utility scores from 15-item self-complete health status questionnaires. 2000.

32. Zigmond AS, Snaith RP. The hospital anxiety and depression scale. Acta Psychiatr Scand. 1983;67(6):361-70. PubMed PMID: 6880820.

33. Wohlfarth TD, van den Brink W, Winkel FW, ter Smitten M. Screening for Posttraumatic Stress Disorder: an evaluation of two self-report scales among crime victims. Psychol Assess. 2003;15(1):101-9. PubMed PMID: 12674729.

34. Kendrick D, Vinogradova Y, Coupland C, Christie N, Lyons RA, Towner EL, et al. Getting back to work after injury: the UK Burden of Injury multicentre longitudinal study. BMC Public Health. 2012;12:584. doi: 10.1186/1471-2458-12-584. PubMed PMID: 22853715; PubMed Central PMCID: PMCPMC3444403.
35. Ahmed W, Alwe R, Wade D. One-year functional outcomes following major trauma: experience of a UK level 1 major trauma centre. Clin Rehabil. 2017;31(12):1646-52. doi: 10.1177/0269215517712044. PubMed PMID: 28580790.
36. Polinder S, Haagsma JA, Belt E, Lyons RA, Erasmus V, Lund J, et al. A systematic review of studies measuring health-related quality of life of general injury populations. BMC Public Health. 2010;10:783. doi: 10.1186/1471-2458-10-783. PubMed PMID: 21182775; PubMed Central PMCID: PMCPMC3019196.
37. Polinder S, Haagsma JA, Bonsel G, Essink-Bot ML, Toet H, van Beeck EF. The measurement of long-term health-related quality of life after injury: comparison of EQ-5D and the health utilities index. Inj Prev. 2010;16(3):147-53. doi: 10.1136/ip.2009.022418. PubMed PMID: 20570982.
38. Manuel DG, Schultz SE, Kopec JA. Measuring the health burden of chronic disease and injury using health adjusted life expectancy and the Health Utilities Index. J Epidemiol Community Health. 2002;56(11):843-50. PubMed PMID: 12388577; PubMed Central PMCID: PMCPMC1732044.
39. Nguyen H, Ivers R, Pham C. Health-related quality of life and recovery patterns among hospitalised injury patients in Vietnam. Qual Life Res. 2018;27(3):619-29. doi: 10.1007/s11136-017-1724-0. PubMed PMID: 29079888.
40. Gabbe BJ, Lyons RA, Sutherland AM, Hart MJ, Cameron PA. Level of agreement between patient and proxy responses to the EQ-5D health questionnaire 12 months after injury. J Trauma Acute Care Surg. 2012;72(4):1102-5.
41. Van Beeck EF, Larsen CF, Lyons RA, Meerding WJ, Mulder S, Essink-Bot ML. Guidelines for the conduction of follow-up studies measuring injury-related disability. J Trauma. 2007;62(2):534-50. doi: 10.1097/TA.0b013e31802e70c7. PubMed PMID: 17297349.

42. Lamers LM, McDonnell J, Stalmeier PF, Krabbe PF, Busschbach JJ. The dutch tariff: Results and arguments for an effective design for national EQ-5D valuation studies. Health Econ 2006;15(10):1121-32.

43. Committee on Injury Scaling, Association for the Advancement of Automotive Medicine (AAAM). The Abbreviated Injury Scale, 1990 Revision. 2008.

44. SociaalenCultureelPlanbureau. Veelgestelde vragen over statusscore, versie 5 maart 2015.

45. van Buuren S, Oudshoorn K. Flexible multivariate imputation by MICE. Leiden: Netherlands Organization for Applied Scientific Research (TNO) Prevention and Health, 1999.

46. Laird NM, Ware JH. Random-effects models for longitudinal data. Biometrics. 1982;38(4):963-74. PubMed PMID: 7168798.

47. Stolk E, Krabbe P, Busschbach J. Using the Internet to collect EQ-5D norm scores: a valid alternative? 24th Scientific Plenary Meeting of the EuroQoL Group - Proceedings: 153-1652009.

48. . Available from: https://www.volksgezondheidenzorg.info/onderwerp/posttraumatische-stressstoornis/cijfers-context/huidige-situatie#node-prevalentie-van-ptss-de-bevolking.

49. Toien K, Bredal IS, Skogstad L, Myhren H, Ekeberg O. Health related quality of life in trauma patients. Data from a one-year follow up study compared with the general population. Scand J Trauma Resusc Emerg Med. 2011;19:22. doi: 10.1186/1757-7241-19-22. PubMed PMID: 21477280; PubMed Central PMCID: PMCPMC3088534.

50. Verweij G, Houben-van Herten M. Depressiviteit en antidepressiva in Nederland, bevolkingstrends 2013. CBS, December 2013.

51. de Graaf R, ten Have M, van Dorsselaer S. De psychische gezondheid van de Nederlandse bevolking. NEMESIS-2: Opzet en eerste resultaten. Utrecht: Trimbos-insituut. Netherlands Institute of Mental Health and Addiction, 2010.

52. Haagsma JA, Polinder S, Toet H, Panneman M, Havelaar AH, Bonsel GJ, et al. Beyond the neglect of psychological consequences: post-traumatic stress disorder increases the non-fatal burden of injury by more than 50%. Inj Prev. 2011;17(1):21-6. doi: 10.1136/ip.2010.026419. PubMed PMID: 20929853.

53. Zarzaur BL, Bell T. Trajectory subtypes after injury and patient-centered outcomes. J Surg Res. 2016;202(1):103-10. doi: 10.1016/j.jss.2015.12.038. PubMed PMID: 27083954; PubMed Central PMCID: PMCPMC4834145.
54. Soberg HL, Bautz-Holter E, Finset A, Roise O, Andelic N. Physical and mental health 10 years after multiple trauma: A prospective cohort study. J Trauma Acute Care Surg. 2015;78(3):628-33. doi: 10.1097/TA.0000000000000541. PubMed PMID: 25710437.
55. Revicki DA, Cella DF. Health status assessment for the twenty-first century: item response theory, item banking and computer adaptive testing. Qual Life Res. 1997;6(6):595-600. PubMed PMID: 9330558.
56. Hung M, Stuart AR, Higgins TF, Saltzman CL, Kubiak EN. Computerized Adaptive Testing Using the PROMIS Physical Function Item Bank Reduces Test Burden With Less Ceiling Effects Compared With the Short Musculoskeletal Function Assessment in Orthopaedic Trauma Patients. J Orthop Trauma. 2014;28(8):439-43. doi: 10.1097/BOT.0000000000000059. PubMed PMID: 24378399.
57. Hung M, Franklin JD, Hon SD, Cheng C, Conrad J, Saltzman CL. Time for a paradigm shift with computerized adaptive testing of general physical function outcomes measurements. Foot Ankle Int. 2014;35(1):1-7. doi: 10.1177/1071100713507905. PubMed PMID: 24101733.
58. Jayakumar P, Overbeek C, Vranceanu AM, Williams M, Lamb S, Ring D, et al. The use of computer adaptive tests in outcome assessments following upper limb trauma. Bone Joint J. 2018;100-B(6):693-702. doi: 10.1302/0301-620X.100B6.BJJ-2017-1349.R1. PubMed PMID: 29855231.
59. Cubbin C, Smith GS. Socioeconomic inequalities in injury: critical issues in design and analysis. Annu Rev Public Health. 2002;23:349-75. doi: 10.1146/annurev.publhealth.23.100901.140548. PubMed PMID: 11910067.
60. Haagsma JA, Maertens de Noordhout C, Polinder S, Vos T, Havelaar AH, Cassini A, et al. Assessing disability weights based on the responses of 30,660 people from four European countries. Popul Health Metr. 2015;13:10. doi: 10.1186/s12963-015-0042-4. PubMed PMID: 26778920; PubMed Central PMCID: PMCPMC4715333.

61. MacKenzie EJ, Siegel JH, Shapiro S, Moody M, Smith RT. Functional recovery and medical costs of trauma: an analysis by type and severity of injury. J Trauma. 1988;28(3):281-97. PubMed PMID: 3351987.

62. Holtslag HR, van Beeck EF, Lindeman E, Leenen LP. Determinants of long-term functional consequences after major trauma. J Trauma. 2007;62(4):919-27. doi: 10.1097/01.ta.0000224124.47646.62. PubMed PMID: 17426549.

63. Kendrick D, Dhiman P, Kellezi B, Coupland C, Whitehead J, Beckett K, et al. Psychological morbidity and return to work after injury: multicentre cohort study. Br J Gen Pract. 2017;67(661):e555-e64. doi: 10.3399/bjgp17X691673. PubMed PMID: 28630058; PubMed Central PMCID: PMCPMC5519127.

64. Wilson SJ, Davie G, Derrett S. Two years after injury: prevalence and early post-injury predictors of ongoing injury-related problems. Qual Life Res. 2017;26(7):1831-8. doi: 10.1007/s11136-017-1521-9. PubMed PMID: 28229327.

65. Richmond TS, Kauder D, Hinkle J, Shults J. Early predictors of long-term disability after injury. Am J Crit Care. 2003;12(3):197-205. PubMed PMID: 12751393.

66. Jacoby SF, Shults J, Richmond TS. The effect of early psychological symptom severity on long-term functional recovery: A secondary analysis of data from a cohort study of minor injury patients. Int J Nurs Stud. 2017;65:54-61. doi: 10.1016/j.ijnurstu.2016.11.004. PubMed PMID: 27863295; PubMed Central PMCID: PMCPMC5148666.

67. Haagsma JA, Graetz N, Bolliger I, Naghavi M, Higashi H, Mullany EC, et al. The global burden of injury: incidence, mortality, disability-adjusted life years and time trends from the Global Burden of Disease study 2013. Inj Prev. 2016;22(1):3-18. doi: 10.1136/injuryprev-2015-041616. PubMed PMID: 26635210; PubMed Central PMCID: PMCPMC4752630.

Compliance with ethical standards

Ethical approval and informed consent: The BIOS was approved by the Medical Ethics Committee Brabant, the Netherlands (project number NL50258.028.14 and NW2016-09). This study was conducted according to the 1964 Declaration of Helsinki and its later amendements. Informed consent was obtained from all individual participants included in the study.

Availability of data and material: Data of the BIOS are unsuitable for public deposition due to the privacy of participant data. Data are anonymized, but due to relatively few severe cases, patients could be identified. Therefore, BIOS data are available for any interested researcher who meets the criteria for access to confidential data. The Brabant Trauma Registry (e-mail: secretariaat@nazb.nl) may be contacted to request data.

Competing interests: The authors declare that there is no conflict of interest.

Author's contributions:

NK: conception and design, acquisition of data, analysis and interpretation of data, drafting the article, critical revision of the article, final approval of the version to be published

SP: conception and design, analysis and interpretation of data, drafting the article, critical revision of the article, final approval of the version to be published

LM: conception and design, acquisition of data, analysis and interpretation of data, drafting the article, critical revision of the article, final approval of the version to be published

CR: conception and design, acquisition of data, analysis and interpretation of data, critical revision of the article, final approval of the version to be published

KL: conception and design, analysis and interpretation of data, final approval of the version to be published

MJ: conception and design, analysis and interpretation of data, drafting the article, critical revision of the article, final approval of the version to be published

BIOS-group: conception and design, final approval of the version to be published

Supporting information

S1 Table. Injury group classification of the most common types of injury, based on the Abbreviated Injury Score [67].

S2 File. Methods of imputed data of the Brabant Injury Outcome Surveillance.

S3 Table. Missing sum scores of the original data and imputed data of the self-reported health status and psychological measures of the participants of the Brabant Injury Outcome Surveillance (n=4,883).

737

738

Rebuttal letter

Response to comments

Additional Editor Comments
-The additional explanation of SES needs to describe whether higher or lower scores indicate greater disadvantage to avoid potential mis-interpretation.
A: In the 'statistical analyses' paragraph, we described the following sentence: a lower status score indicates a lower SES whereas a higher status score indicates a higher SES. Furthermore, we described in the discussion and conclusion paragraphs that a lower status scores is an indicator of a lower SES-level (lines 425 and 473).

-The axes on Figure 5 are not clear. Which data do the titles on the left and right-hand x axes correspond to? Moreover, are these truly demonstrating "differences" or mean values? Ideally it is a good idea to depict variability (e.g., sd, se or 95%CI around the mean).
A: We changed Figure 3 as supposed by the editor. Furthermore, we made the description of the y-axis more clear.

-Supplementary Figure 5 does not appear to have been uploaded.
A: In this manuscript, there is no supplementary Figure 5. In the text, we did not refer to Supplementary Figure 5.

-The explanation of how to interpret a beta score is not sufficient. I suggest you follow the recommendation of Reviewer 2 in providing a clearer explanation of the beta value.
A: See the comment of reviewer 2: we have changed the sentence as supposed by the reviewer.

Comments reviewer 1
-There is still some language editing required which can be done at the proofing stage if the manuscript is accepted by the journal.
A: We did made several improvements in language editing throughout the manuscript.

-One sentence that is important to address in the abstract is: 'We aimed to describe the recovery patterns of health status (HS) and psychological outcomes during 24 months of follow-up and to identify subgroups at risk of both short and long-term HS after trauma'. The authors should make it clear that the focus is on identification of sub-groups at risk of poor health status.
A: In the abstract and manuscript, we now described: 'We aimed to 1) identify subgroups at risk of both short and long-term health status (HS) after trauma and 2) to describe the recovery patterns of HS and psychological outcomes during 24 months of follow-up.'

Comments reviewer 2
-The manuscript still requires significant editing for grammatical and spelling errors. For example, in line 71, no comma is required; line 106 should read 'compiles' and not 'complies'; line 140 should read 'comprise' and not 'comproise'. Errors are too extensive to list them all. Spelling/gramma correction software should help (not just the Word tool).
A: We have deleted the comma in line 71, we have changed the word 'complies' into 'compiles' and changed the word 'comproise' into 'comprise'. Furthermore, we made several improvements in language editing throughout the manuscript.

-Line 108. Please explain 'randomly controlled the data' in the data verification process more detail. All patient files or a sample? Did you check completeness, consistency, coherence and/or chronology?

A: We now described the following sentence: 'Quality of the data of the BTR and BIOS was checked on outliers and completeness by a trauma coordinator and researcher respectively. Furthermore, data from a sample of the trauma registry was checked manually by a trauma surgeon.'

-Line 240. Spell out FU. Again, using too many acronyms that are not widely recognized makes reading very arduous
A: We have spelled out the FU as 'follow-up' as suggested by the reviewer.

-Table 4 'Beta: measures how strong each predictor variable influences the dependent variable' could be replaced by 'mean increase in EQ-5D-3L score (improvement in quality of life) compared to the reference category.
A: We thank the reviewer for this suggestion. We changed the sentence as supposed by the reviewer.

Comments reviewer 3
-I am still a little confused by the status-scores.
A: In the 'statistical analyses' paragraph, we described the following sentence: a lower status score indicates a lower SES whereas a higher status score indicates a higher SES. Hopefully, this will lead to a better interpretation of the status score. Furthermore, we described in the discussion and conclusion paragraphs that a lower status scores is an indicator of a lower SES-level (lines 425 and 473). To interpret the results, we added the minimum and maximum scores.

-Some of the confidence intervals contains negative figures. What are the extremes of the scale? As this seems to be a Dutch scale, you could explain this better to international readers.
A: We added the minimum and maximum score of the EQ-5D-3L under Table 4.

-In the study design and participants section it is stated: "The Brabant Trauma Registry (BTR) complies pre-hospital and hospital data of all trauma patients admitted after presentation to the ED in the Noord-Brabant region." You probably intended to say compiles?
A: Indeed, we intented to say 'compile' instead of 'complies'. We changed the word 'complies' into 'compiles'.

Same paragraph: "Before the data of the BTR and data of the BIOS-study were merged, the researchers randomly controlled the data of the trauma registry." This is commendable, but what was "randomly"? 1 of 100, 1 of 1000, 20%? Please elaborate, otherwise this statement is not very descriptive.
A: We deleted the following text: 'Before the data of the BTR and data of the BIOS-study were merged, the researchers randomly controlled the data of the trauma registry'. We replaced this text with the following sentence: 'Quality of the data of the BTR and BIOS was checked on outliers and completeness by a trauma coordinator and researcher respectively. Furthermore, data from a sample of the trauma registry was checked manually by a trauma surgeon.'
