# Supplementary material for: Health status and psychological outcomes after trauma: A prospective multicenter cohort study
Source: PLoS One. 2020 Apr 21;15(4):e0231649. doi: 10.1371/journal.pone.0231649 (PMC7173764; doi:10.1371/journal.pone.0231649)
Supplement: Attachment — Submitted filename: Response to Reviewers_V2_08032020(2).docx [file pone.0231649.s005.docx]

**Rebuttal letter**

**Response to comments**

**Additional Editor Comments**

-The additional explanation of SES needs to describe whether higher or lower scores indicate greater disadvantage to avoid potential mis-interpretation.

*A: In the ‘statistical analyses’ paragraph, we described the following sentence: a lower status score indicates a lower SES whereas a higher status score indicates a higher SES. Furthermore, we described in the discussion and conclusion paragraphs that a lower status scores is an indicator of a lower SES-level (lines 425 and 473).*

-The axes on Figure 5 are not clear. Which data do the titles on the left and right-hand x axes correspond to? Moreover, are these truly demonstrating "differences" or mean values? Ideally it is a good idea to depict variability (e.g., sd, se or 95%CI around the mean).

*A: We changed Figure 3 as supposed by the editor. Furthermore, we made the description of the y-axis more clear.*

-Supplementary Figure 5 does not appear to have been uploaded.

*A: In this manuscript, there is no supplementary Figure 5. In the text, we did not refer to Supplementary Figure 5.*

-The explanation of how to interpret a beta score is not sufficient. I suggest you follow the recommendation of Reviewer 2 in providing a clearer explanation of the beta value.

*A: See the comment of reviewer 2: we have changed the sentence as supposed by the reviewer.*

**Comments reviewer 1**

-There is still some language editing required which can be done at the proofing stage if the manuscript is accepted by the journal.

*A: We did made several improvements in language editing throughout the manuscript.*

-One sentence that is important to address in the abstract is: ‘We aimed to describe the recovery patterns of health status (HS) and psychological outcomes during 24 months of follow-up and to identify subgroups at risk of both short and long-term HS after trauma’. The authors should make it clear that the focus is on identification of sub-groups at risk of poor health status.

*A: In the abstract and manuscript, we now described: ‘We aimed to 1) identify subgroups at risk of both short and long-term health status (HS) after trauma and 2) to describe the recovery patterns of HS and psychological outcomes during 24 months of follow-up.’*

**Comments reviewer 2**

-The manuscript still requires significant editing for grammatical and spelling errors. For example, in line 71, no comma is required; line 106 should read ‘compiles’ and not ‘complies’; line 140 should read ‘comprise’ and not ‘comproise’. Errors are too extensive to list them all. Spelling/gramma correction software should help (not just the Word tool).

*A: We have deleted the comma in line 71, we have changed the word ‘complies’ into ‘compiles’ and changed the word ‘comproise’ into ‘comprise’. Furthermore, we made several improvements in language editing throughout the manuscript.*

-Line 108. Please explain ‘randomly controlled the data’ in the data verification process more detail. All patient files or a sample? Did you check completeness, consistency, coherence and/or chronology?

*A: We now described the following sentence: ‘Quality of the data of the BTR and BIOS was checked on outliers and completeness by a trauma coordinator and researcher respectively. Furthermore, data from a sample of the trauma registry was checked manually by a trauma surgeon.’*

-Line 240. Spell out FU. Again, using too many acronyms that are not widely recognized makes reading very arduous
*A: We have spelled out the FU as ‘follow-up’ as suggested by the reviewer.*

-Table 4 ‘Beta: measures how strong each predictor variable influences the dependent variable’ could be replaced by ‘mean increase in EQ-5D-3L score (improvement in quality of life) compared to the reference category.

*A: We thank the reviewer for this suggestion. We changed the sentence as supposed by the reviewer.*

**Comments reviewer 3**

-I am still a little confused by the status-scores.

*A: In the ‘statistical analyses’ paragraph, we described the following sentence: a lower status score indicates a lower SES whereas a higher status score indicates a higher SES. Hopefully, this will lead to a better interpretation of the status score. Furthermore, we described in the discussion and conclusion paragraphs that a lower status scores is an indicator of a lower SES-level (lines 425 and 473). To interpret the results, we added the minimum and maximum scores.*

-Some of the confidence intervals contains negative figures. What are the extremes of the scale? As this seems to be a Dutch scale, you could explain this better to international readers.

*A: We added the minimum and maximum score of the EQ-5D-3L under Table 4.*

-In the study design and participants section it is stated: “The Brabant Trauma Registry (BTR) complies pre-hospital and hospital data of all trauma patients admitted after presentation to the ED in the Noord-Brabant region.” You probably intended to say compiles?

*A: Indeed, we intented to say ‘compile’ instead of ‘complies’. We changed the word ‘complies’ into ‘compiles’.*

Same paragraph: “Before the data of the BTR and data of the BIOS-study were merged, the researchers randomly controlled the data of the trauma registry.” This is commendable, but what was “randomly”? 1 of 100, 1 of 1000, 20%? Please elaborate, otherwise this statement is not very descriptive.

*A: We deleted the following text: ‘Before the data of the BTR and data of the BIOS-study were merged, the researchers randomly controlled the data of the trauma registry’. We replaced this text with the following sentence: ‘Quality of the data of the BTR and BIOS was checked on outliers and completeness by a trauma coordinator and researcher respectively. Furthermore, data from a sample of the trauma registry was checked manually by a trauma surgeon.’*
