# Supplementary material for: Health status and psychological outcomes after trauma: A prospective multicenter cohort study
Source: PLoS One. 2020 Apr 21;15(4):e0231649. doi: 10.1371/journal.pone.0231649 (PMC7173764; doi:10.1371/journal.pone.0231649)
Supplement: Attachment — Submitted filename: Rebuttal letter_BIOS_V3_27032020.docx [file pone.0231649.s007.docx]

**Rebuttal letter**

**Response to comments**

-In Fig. 3, we changed the word ‘’sumscore’’ into ‘’summary score’’, as suggested by the editor.

-In the abstract and in the introduction, we changed the description of the aim of the study, as suggested by the editor.

-In line 111, we added the following information on how data was checked: ‘Data of the BTR (i.e. Brabant Trauma Registry) was checked as part of routine practice while the data of the BIOS was checked randomly by the researchers.’

-In line 140, we removed the question mark as supposed by the editor.

-In one of the previous versions of our submitted manuscript, we referred to S5. This was not correct, we should have referred to Fig 3 instead of S5. In the current manuscript, we now correctly refer to Fig 3.

-In line 398, we removed the ‘’s’’ as supposed by the editor.

-In line 457, we removed the ‘’n’’ as supposed by the editor.
